# NAT10 inhibition alleviates renal tubular epithelial cell senescence by impeding ac4C acetylation of *PAPP-A* mRNA in diabetic nephropathy

**DOI:** 10.1038/s41419-026-08970-0

**Published:** 2026-06-11

**Authors:** Mingyang Hu, Linxiao Lv, Ruxu Li, Zihao Zhao, Wenxin Li, Yanyan Wang, Zhangsuo Liu, Yanfang Lu, Sijie Zhou

**Affiliations:** 1 Department of Nephrology, The First Affiliated Hospital of Zhengzhou University, Zhengzhou, China; 2 Research Institute of Nephrology, Zhengzhou University, Zhengzhou, China; 3 Traditional Chinese Medicine Integrated Department of Nephrology, The First Affiliated Hospital of Zhengzhou University, Zhengzhou, China; 4 Tianjian Laboratory of Advanced Biomedical Sciences, Academy of Medical Sciences, Zhengzhou University, Zhengzhou, China

**Keywords:** Molecular biology, Epigenetics

## Abstract

Cellular senescence plays a critical role in diabetic nephropathy (DN). Understanding the mechanisms underlying the senescence response is therefore essential for developing effective therapies for DN. In this study, we provide evidence that NAT10 expression was markedly elevated in the kidney tissues of patient with DN and was associated with adverse clinical outcomes. NAT10 directly bound to PAPP-A, induced ac4C acetylation modification of the PAPP-A transcript, and enhanced its stability, thereby promoting PAPP-A upregulation. Moreover, NAT10-mediated ac4C acetylation accelerated renal tubular epithelial cell senescence in DN through the PAPP-A/p53 pathway. Conditional knockout (cKO) of NAT10 attenuates renal tubular senescence in streptozotocin (STZ)-induced type 1 diabetes mellitus (T1DM) mouse models. Renal tubular-specific knockdown of NAT10 via targeted adeno-associated virus ameliorates renal tubular epithelial cell senescence in db/db mouse models of type 2 diabetes mellitus (T2DM). Pharmacological inhibition of NAT10 with Remodelin protects against renal tubular epithelial cell senescence both in vivo and in vitro. In conclusions, NAT10-mediated ac4C acetylation contributes to renal tubular epithelial cell senescence in DN and that NAT10-regulated PAPP-A could be a promising and feasible therapeutic target in DN.

**Figure:**
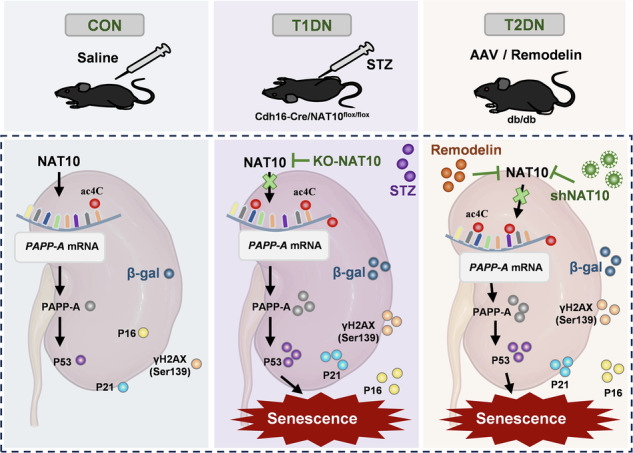
A schematic diagram illustrates the multifaceted role of the NAT10 in renal tubular epithelial cell senescence upon type 1 and type 2 diabetic insult and the protective effect of targeting NAT10. NAT10 is currently recognized as the sole “writer” enzyme responsible for catalyzing the ac4C modification. The present study demonstrated that NAT10 is predominantly expressed in renal tubule and highly expressed in DN and correlated with renal tubular epithelial cell senescence. NAT10-mediated ac4C modification enhanced the expression of PAPP-A, which may directly influence senescence signaling by interacting with the key mediator p53, thereby promoting senescence signaling and renal tubular epithelial cell senescence. The aforementioned pathogenic pathways contribute to diabetic renal tubular cell injury in both type 1 and type 2 diabetes and can be mitigated by targeting NAT10, either genetically through conditional knockout (cKO) or adeno-associated virus-mediated knockdown, or pharmacologically using small-molecule inhibitors such as Remodelin.

A schematic diagram illustrates the multifaceted role of the NAT10 in renal tubular epithelial cell senescence upon type 1 and type 2 diabetic insult and the protective effect of targeting NAT10. NAT10 is currently recognized as the sole “writer” enzyme responsible for catalyzing the ac4C modification. The present study demonstrated that NAT10 is predominantly expressed in renal tubule and highly expressed in DN and correlated with renal tubular epithelial cell senescence. NAT10-mediated ac4C modification enhanced the expression of PAPP-A, which may directly influence senescence signaling by interacting with the key mediator p53, thereby promoting senescence signaling and renal tubular epithelial cell senescence. The aforementioned pathogenic pathways contribute to diabetic renal tubular cell injury in both type 1 and type 2 diabetes and can be mitigated by targeting NAT10, either genetically through conditional knockout (cKO) or adeno-associated virus-mediated knockdown, or pharmacologically using small-molecule inhibitors such as Remodelin.

## Introduction

Diabetic nephropathy (DN) is a major global cause of end-stage renal disease (ESRD) and represents a substantial public health burden, profoundly diminishing patients’ quality of life [1, 2]. DN develops in roughly 30% of individuals with type 1 diabetes mellitus (T1DM) and up to 40% of those with type 2 diabetes mellitus (T2DM) [3]. Current therapeutic options for DN only attenuate disease progression but fail to halt its onset or advancement [4]. Therefore, elucidating the underlying mechanisms of DN pathogenesis is crucial for developing novel therapeutic targets and strategies to improve clinical outcomes.

Although DN has traditionally been viewed as a glomerular disorder [5, 6], growing evidence indicates that renal tubular injury is equally pivotal to its pathogenesis [7, 8]. Proximal tubular injury markers correlate strongly with DN progression, independent of classical glomerular indices such as albuminuria [9]. Patients with type 2 DN frequently display an accelerated senescent phenotype in tubular epithelial cells [10], and heightened cellular senescence has been consistently observed in tubular compartments of individuals with diabetes and DN [10], implicating senescence-associated pathways in disease evolution. A substantial body of evidence demonstrates that accelerated renal tubular cell senescence is a central cellular event in the onset and progression of DN [11–13]. Nonetheless, the mechanisms driving renal tubular cell senescence in DN remain poorly understood and require further investigation.

Epigenetic modifications of mRNA are increasingly recognized as critical regulators of kidney disease initiation and progression. Among these, N4-acetylcytidine (ac4C) is a naturally occurring RNA modification that is pivotal for maintaining mRNA stability [14–16], post-transcriptional regulation, and translation efficiency [17]. N-acetyltransferase 10 (NAT10) is the sole known acetyltransferase that catalyzes ac4C deposition. Increasing evidence indicates that aberrant regulation of NAT10 and ac4C modifications participates in multiple senescence-associated processes, including DNA damage repair [18], and cell cycle regulation [19]. Although recent studies have identified the pathological roles of NAT10 in renal cell inflammation [20] and ferroptosis [21] in acute kidney injury, the mechanisms and functions of NAT10-mediated ac4C modification in DN remain largely unclear.

In this study, we delineate the mechanistic and functional roles of NAT10 in DN and renal cellular senescence. We found that NAT10 expression was markedly increased in kidney biopsy specimens from patients with DN and closely associated with renal tubular epithelial cell senescence. Mechanistically, NAT10-mediated ac4C modification enhanced the stability of *pregnancy associated plasma protein A (PAPP-A)* mRNA and promoted renal tubular epithelial cell senescence in DN through the PAPP-A/p53 pathway. Notably, both genetic and pharmacological suppression of NAT10 reduced PAPP-A expression and protected against renal tubular epithelial cell senescence in experimental DN. These findings provide a molecular basis for targeting NAT10-dependent ac4C modification as a potential therapeutic strategy to mitigate renal tubular epithelial cell senescence and DN.

## Materials and method

### Patient specimens

All human specimens were obtained from the First Affiliated Hospital of Zhengzhou University in compliance with the Declaration of Helsinki and with approval from the institutional ethics committee (No. 2023-KY-0656-002). Kidney tissues for the DN group were provided by the Department of Nephrology. The inclusion criteria for the DN samples were as follows: (1) patients with a clinical diagnosis of DN and (2) patients without malignancies, infections, or other renal disorders. Kidney tissues from 14 patients who underwent total or partial nephrectomy for non-diabetic conditions in the Department of Renal Transplantation were used as negative controls.

### Animals

All animal procedures were approved by the Ethics Committee of the Experimental Animal Center of Zhengzhou University (License No. ZZU-LAC20230616(06)). All experiments were conducted in accordance with the National Institutes of Health (NIH) guidelines for the care and use of laboratory animals [22]. The mice were housed and maintained at the Experimental Animal Center of Zhengzhou University.

### Mouse type 2 diabetic model

Male db/db and db/m mice, eight weeks of age, were obtained from Cyagen Biosciences, Inc. (Jiangsu, China; Animal Qualification Certificate No. SCXK (Su) 2022-0016). Before initiating the experiments, all animals underwent a one-week acclimation period and were subsequently randomized into the designated experimental groups.

In this study, mice were subjected to either adeno-associated virus (AAV) administration (Hanbio Tech, Shanghai, China) or treatment with the NAT10 inhibitor Remodelin (MedChemExpress, #HY-16706) in separate experimental settings. To assess the impact of NAT10 knockdown on renal aging, animals were allocated into the following groups and received the corresponding AAV vectors: db/m, db/db, db/db injected with a control vector (db/db + shNC), and db/db injected with an NAT10 knockdown vector (db/db + shNAT10). To evaluate the effects of Remodelin on renal tubular epithelial cell senescence, three groups were included: db/m, db/db, and db/db treated with Remodelin (db/db + RH). Remodelin was administered intraperitoneally at 5 mg/kg every other day for a total of 12 weeks.

### Tamoxifen-induced cKO model of NAT10 in renal tubular epithelial cells

In this experiment, 8-week-old uninduced renal tubular epithelial cell-specific *NAT10* conditional knockout (cKO) mice were used. Each mouse was weighed individually to ensure accurate dose calculation. Tamoxifen was dissolved in corn oil (MCE, Cat. No. HY-Y1888, 50 mL) as the solvent. Because tamoxifen is poorly soluble, complete dissolution was achieved through repeated pipetting and vortexing, yielding a stock solution at a concentration of 10 mg/mL. The prepared solution was stored at 4 °C in the dark until use. Tamoxifen was administered intraperitoneally at a dose of 40 mg/kg for 5–6 consecutive days. This procedure successfully induced conditional knockout of the *NAT10* in renal tubular epithelial cells.

### Cell culture transfection and treatment

The HK2 cells utilized in this study were purchased from QuiCell Bioscience, Inc. (Shanghai, China). Cells were cultured in low-glucose DMEM (Thermo Fisher Scientific, #11885084) supplemented with 10% fetal bovine serum, and 1% penicillin was used to cultivate these cells in T25 cell culture flasks. The culture of cells was kept at 37 °C in a humidified incubator with 5% CO₂.

An appropriate number of cells were seeded into six-well plates, followed by lentiviral infection. Transfection efficiency was evaluated using Western blot (WB) and quantitative PCR (qPCR) 48–72 h after infection. The lentivirus exhibiting the highest knockdown efficiency was selected for subsequent experiments. Prior to glucose stimulation, HK-2 cells were serum-starved in serum-free medium for 12 h to synchronize the cells and facilitate the establishment of the senescence model. The glucose concentration in the experimental group was 30 mM, compared with 5.6 mM in the control group, and the stimulation period lasted 48 h.

### Western blot

Prepare the gel required for electrophoresis following the protocol specified by the reagent manufacturer. To each well, 20 µg of protein sample was added. Continue electrophoresing until the gel’s bottom is reached. The proteins were placed at 250 mA for 90 minutes on a polyvinylidene fluoride membrane (Millipore, #IPVH00010). After blocking the membrane with 5% skim milk, it was washed three times for 5 minutes each using TBST (Epizyme Biotech, #TF103). After adding the primary antibody, it was incubated at 4 °C throughout the night. The next day, the membrane was incubated with secondary antibody for an hour at room temperature. Enhanced chemiluminescence on a Bio-Rad ChemiDoc XRS+ imaging system was used to visualize protein bands. The Supplementary Material displays three biological replicate bands as well as uncropped WB bands.

### RT-PCR

The following primer sequences were employed in the qPCR: *NAT10* (forward, GACCACGACGACAGCCAGATTG; reverse: ATCCAGGCACAGCAAGTCATTAG); *PAPP-A* (forward: TCTGCTGGACACGAGTCTGGAG; reverse: CACCTTGGGCTGGCGGAAAC); *β-actin* (forward, CACTCTTCCAGCCTTCCTTC; reverse, GTACAGGTCTTTGCGGATGT).

RNA was extracted using the TRIzol reagent according to the manufacturer’s protocol. cDNA was synthesized from the extracted RNA using a reverse transcription kit (TOYOBO, #FSQ - 101), following the provided protocol. Then, the cDNA, forward and reverse primers, and SYBR (TOYOBO, #QPX - 201) were added to prepare a 20-μL reaction system for PCR. β-actin was used as an internal reference.

### ac4C dot blot

Total RNA was isolated from renal tissues using TRIzol reagent and subsequently purified with the Dynabeads™ mRNA Purification Kit. Purified mRNA was denatured by heating at 65 °C for 5 min and rapidly chilled on ice for 5 min. Equal amounts of denatured mRNA were then applied onto activated Hybond-N⁺ membranes, followed by UV cross-linking at 254 nm (150 mJ/cm²). After cross-linking, the membranes were blocked with 5% nonfat milk for 1 h and incubated overnight at 4 °C with an anti-ac4C antibody (Abcam, ab252215). The next day, the primary antibody was removed, and the membranes were washed and incubated with an anti-rabbit IgG secondary antibody (1:5000 dilution) at 37 °C for 1 h. To confirm equal RNA loading, the membranes were briefly stained with 0.2% methylene blue. Signal detection was performed using a chemiluminescent imaging system.

### acRIP sequencing

The acRIP-seq library construction steps were as follows: (1) mRNA was enriched using oligo d(T) magnetic beads; (2) mRNA were broken into ~100-bp short fragments with divalent cations; (3) Immunoprecipitation was performed with an ac4C antibody; (4) First-strand cDNA was synthesized with random hexanucleotide primers using the IP-obtained RNA as the template; (5) The splint adapter is connected; (6) The adapter-ligated product was purified using magnetic beads and PCR amplification was conducted; (7) The library quality was checked by agarose gel electrophoresis; (8) The library was quantified using Qubit 3.0 to check whether its concentration was suitable for sequencing; (9) After quality check, load sequencing libraries according to target data volume and effective concentration.

After obtaining the raw sequencing data, filter it first to obtain clean data. Align the clean data to the project species’ reference genome to get comprehensive transcript info, and conduct gene expression quantification, GO, KEGG Pathway analyses, etc. The data have been deposited in the Gene Expression Omnibus (GEO) database (Accession: GSE330937).

### Statistical analysis

All experiments were performed in at least three independent replicates. Statistical analyses were conducted using GraphPad Prism v8. Differences between groups were assessed using one-way ANOVA or two-tailed unpaired Student’s *t* tests, as appropriate. A *p* value of <0.05 was considered statistically significant.

## Results

### Renal tubular epithelial cell senescence is associated with NAT10 overexpression in DN

**Fig. 1:**
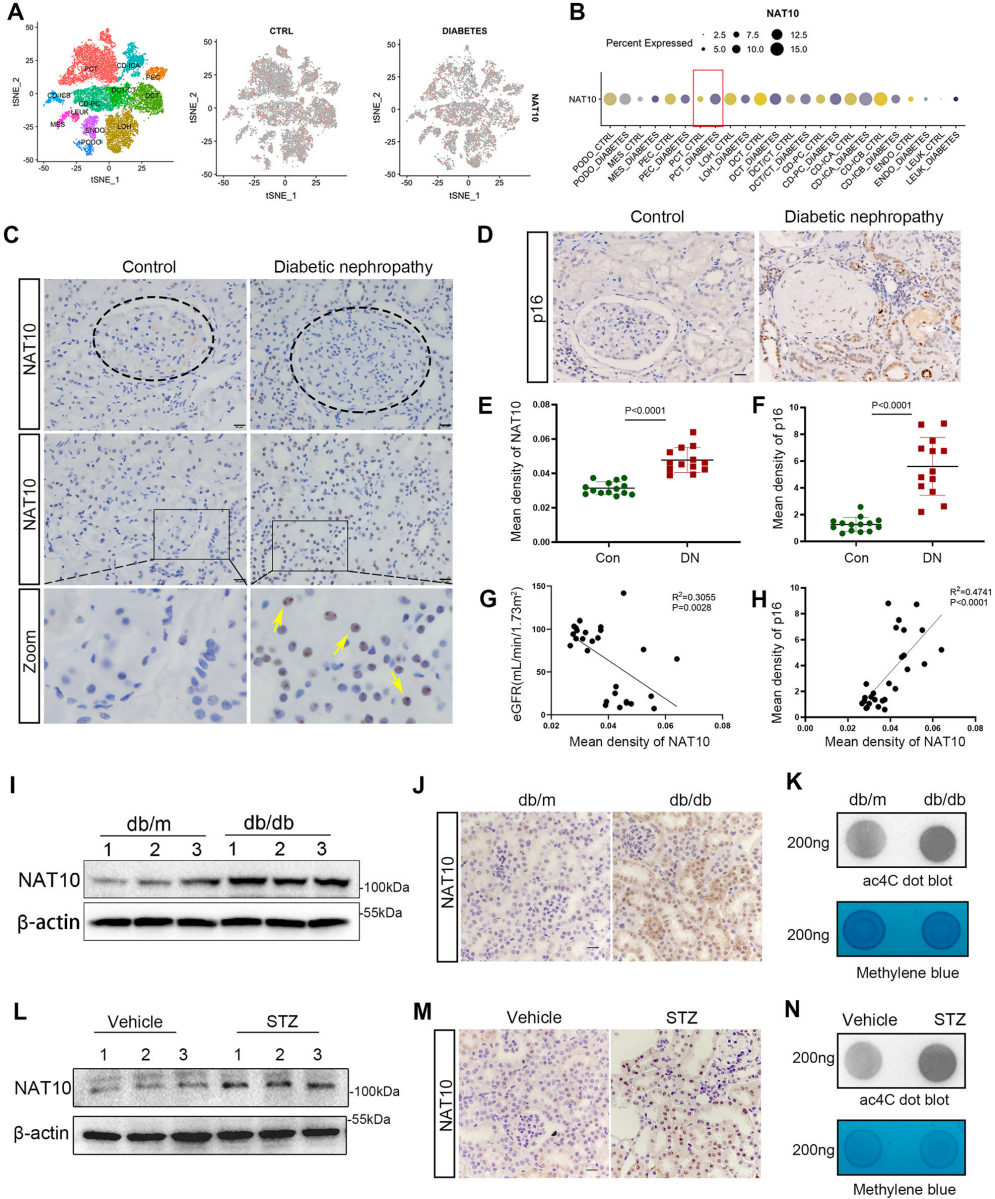
NAT10 expression and ac4C modifications are elevated in diabetic nephropathy. **A** Diabetic nephropathy and control samples were integrated into a single dataset and clustered using Seurat. The following abbreviations are used: PCT proximal convoluted tubule, CFH complement factor H, LOH loop of Henle, DCT distal convoluted tubule, CT connecting tubule, CD collecting duct, PC principal cell, IC intercalated cell, PODO podocyte, ENDO endothelium, MES mesangial cell, and LEUK leukocyte. The distribution of NAT10 in the single-cell transcriptomic landscape. **B** The distribution of NAT10 in different cells in different groups. **C** Immunohistochemistry was utilized to detect the expression of NAT10 in kidney sections of patients with DN. The scale bars = 20 μm. **D** Immunohistochemistry was utilized to detect the expression of p16 in kidney sections of patients with DN. The scale bars = 20 μm. **E** The mean density analysis of NAT10. **F** The mean density analysis of p16. **G** Correlation analysis between eGFR and NAT10. **H** Correlation analysis between p16 and NAT10. **I** Western blotting analyses of NAT10 in db/db mice. **J** NAT10 staining in kidney sections of db/db mice. **K** The ac4C level in db/db mice was determined using ac4C dot blot assay. **L** Western blotting analyses of NAT10 in STZ-induced T1DM mice. **M** NAT10 staining in kidney sections of STZ-induced T1DM mice. **N** The ac4C level in STZ-induced T1DM mice was determined using ac4C dot blot assay.

To investigate whether NAT10-mediated ac4C acetylation is involved in DN, we analyzed the single-cell transcriptomic atlas of early-stage human DN using the public Kidney Interactive Transcriptomics (KIT) database (https://humphreyslab.com/SingleCell/), as reported by Wilson et al. [23]. We found that patients with early DN exhibited elevated NAT10 expression in renal tubules (Fig. 1A, B). To further validate NAT10 expression and localization in DN, we performed immunohistochemical analyses on renal tissue samples obtained from 27 participants at the First Affiliated Hospital of Zhengzhou University, China. The DN group included renal biopsy specimens from 13 patients, whereas the control group comprised kidney cortex samples from 14 nondiabetic individuals who underwent nephrectomy for renal masses. (Fig. 1C, E). Moreover, correlation analysis with clinical parameters showed that NAT10 was negatively correlated with eGFR, Hb, ALB, and positively correlated with BUN, Scr, β2-MG, Cys-C, UA and T-CHO (Figs. 1G and S1A). In addition, renal tubular injury and epithelial cell senescence were observed in both patients and murine models of DN (Figs. 1D and S1B, C). Computerized morphometric analysis further demonstrated that NAT10 levels were positively correlated with p16 expression in human diabetic kidney biopsy samples (Fig. 1H). Collectively, these findings indicate that NAT10 overexpression is closely associated with renal tubular epithelial cell senescence and the deterioration of kidney function in patients with DN.

Consistent results were observed for NAT10 expression in patients with DN. NAT10 levels were significantly upregulated in both spontaneous type 2 diabetic (db/db) mice and STZ-induced type 1 diabetic mice compared with controls (Fig. 1I, L). The results of immunohistochemical staining were consistent with those obtained from WB analysis (Fig. 1J, M). Notably, ac4C dot blot analysis demonstrated a marked increase in ac4C levels in both db/db mice and STZ-induced diabetic mice (Fig. 1K, N).

### NAT10-mediated ac4C RNA aceytlation enhances *PAPP-A* mRNA stability

**Fig. 2:**
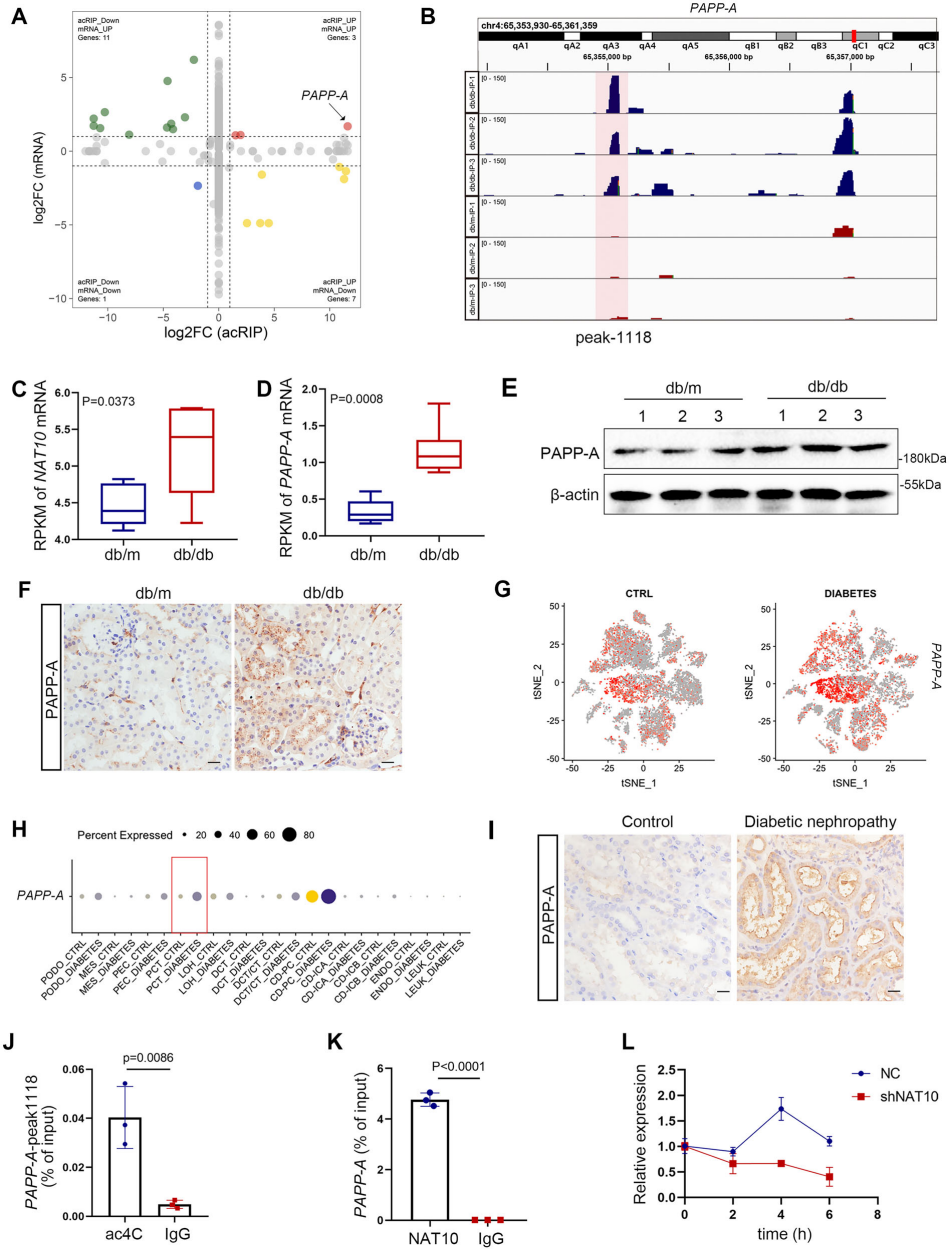
NAT10-mediated ac4C RNA acetylation enhances *PAPP-A* mRNA stability. **A** Volcano plot of the combined analysis of acRIP-seq and RNA-seq in db/m mice and db/db mice. **B** Integrative Genomics Viewer (IGV) tracks displaying the *PAPP-A* ac4C peaks. **C** The expression of *NAT10* in the transcriptome. **D** The expression of *PAPP-A* in the transcriptome. **E** Western blot was used to detect PAPP-A expression in different groups (*n* = 6). **F** Immunohistochemical detection of PAPP-A expression in kidney tissue of db/db Mice. The scale bars = 20 μm. **G** The distribution of *PAPP-A* in the single-cell transcriptomic landscape. **H** The distribution of *PAPP-A* in different cells in different groups. **I** Immunohistochemical detection of PAPP-A expression in renal tissues of patients with DN. **J** acRIP-qPCR was used to test the ac4C modification levels of *PAPP-A*. **K** *NAT10* immunoprecipitation followed by RT-qPCR disclosed that PAPP-A interacted with NAT10 (*n* = 3). **L** The impression of NAT10 on *PAPP-A* mRNA stability was confirmed by the RNA stability assay.

NAT10 is an acetyltransferase that catalyzes the acetylation of proteins and RNA and exerts broad pathological effects through the regulation of mRNA stability. Although three recent studies have identified the pathological role of NAT10 in kidney cancer and acute kidney disease [20, 21, 24], the mechanisms underlying NAT10-mediated ac4C modification in DN remain unknown. To elucidate how NAT10-mediated ac4C modification contributes to renal tubular epithelial cell senescence in DN, we performed ac4C-specific RNA immunoprecipitation followed by next-generation sequencing (acRIP-seq) in a mouse model of DN. Analyses of acRIP-seq and RNA-seq data from db/m and db/db mice revealed that ac4C peaks were predominantly enriched within coding sequences (CDS) and 3′ untranslated regions (Fig. S2A, B). We identified 162 differential ac4C peaks, including 84 that were upregulated and 78 that were downregulated (Fig. S2C). The representative ac4C sequence motifs are shown in Fig. S2E. To define downstream effectors of NAT10, we performed a combined analysis of differentially expressed genes (DEGs) from acRIP–seq and RNA–seq, which yielded three candidates exhibiting concordant increases in both transcript abundance and ac4C modification. Among them, *PAPP-A* showed the most prominent changes (Fig. 2A). The peaks of PAPP-A mRNA ac4C modification on RNA were visualized and identified using IGV software (Fig. 2B).

Both *NAT10* and *PAPP-A* mRNA levels were upregulated in the transcriptomic data (Fig. 2C, D). Western blot and immunohistochemical analyses further demonstrated increased PAPP-A protein expression in the renal tubules of db/db mice (Fig. 2E, F). Additionally, single-cell transcriptomic data from public databases confirmed elevated PAPP-A expression in renal tubules during the early stages of DN (Fig. 2G, H). The expression of PAPP-A in human tissue samples showed a trend consistent with the above mouse models (Fig. 2I). Furthermore, acRIP-PCR analysis verified that the upregulation of PAPP-A-Peak1118 expression was attributable to ac4C modification of its mRNA (Fig. 2J). However, it remains unclear whether the ac4C modification of *PAPP-A* mRNA is regulated by NAT10. Therefore, we enriched total mRNA using the NAT10 antibody, and the results demonstrated that NAT10 may bind to *PAPP-A* mRNA (Fig. 2K). Finally, actinomycin D chase assays demonstrated that silencing NAT10 markedly diminished the stability of PAPP-A mRNA (Fig. 2L). Taken together, these findings indicate that NAT10-dependent ac4C RNA acetylation enhances the stability of *PAPP-A* transcripts, thereby promoting its increased expression.

### NAT10 knockdown mitigates high-glucose–induced cellular senescence in HK-2 cells

**Fig. 3:**
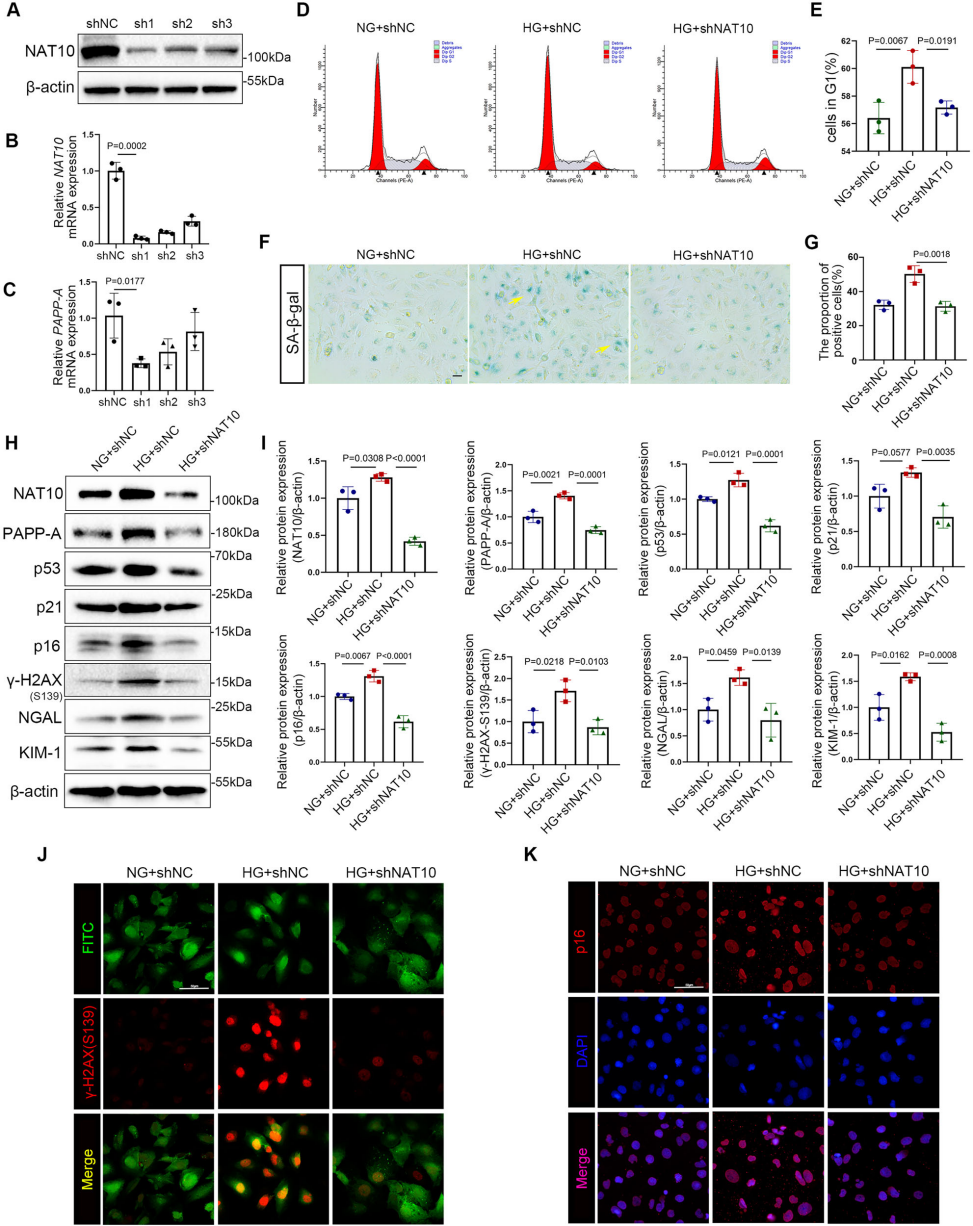
Knockdown of NAT10 reduced cellular senescence in high glucose-stimulated HK-2 cells. **A** Western blot was used to detect the efficiencies of the three knockdown viruses. **B** Detect the efficiencies of the three NAT10-knockdown viruses by RT-PCR **C** Detect the effect of NAT10 knockdown on *PAPP-A* by RT-PCR. **D** Flow cytometry is used to detect the cell cycle changes of different groups. **E** The proportion of cells in the G1 phase for different groups. **F** Representative micrographs showing SA-β-gal staining in different groups. The scale bars = 20 μm. **G** The quantification of the SA-β-gal-positive cells was determined after three biological replicates. **H** Western blot was used to detect NAT10, PAPP-A, p53, p21, p16, NGAL, KIM-1 and γH2AX (S139) expression in different groups (*n* = 3). **I** Densitometric analyses of the expression of NAT10, PAPP-A, p53, p21, p16, NGAL, KIM-1, γH2AX in tissue, presented as relative levels normalized to β-actin levels. **J** Representative micrographs show the expression of γH2AX (S139) in different groups. Red represents γH2AX (S139), Green represents FITC and blue represents DAPI. The scale bars = 50 μm. **K** Representative micrographs show the expression of p16 in different groups. Red represents p16 and blue represents DAPI. The scale bars = 50 μm.

To define the contribution of NAT10 to tubular injury, we silenced *NAT10* in human kidney proximal tubular epithelial (HK-2) cells using short hairpin RNA (shRNA). Efficient knockdown was confirmed by RT–PCR and immunoblot analysis (Fig. 3A–C). Using the sh1 construct, we next assessed cellular senescence by flow cytometry and senescence-associated β-galactosidase (SA-β-gal) staining. Suppression of *NAT10* reduced the proportion of cells arrested in G1 and markedly decreased the number of SA-β-gal–positive cells under high-glucose exposure (Fig. 3D–G).

Immunoblotting further demonstrated that *NAT10* knockdown blunted the high-glucose–induced upregulation of NAT10 and PAPP-A. High-glucose stimulation increased the expression of canonical senescence- and renal tubular injury-associated markers—including p53, p21, p16, KIM-1, NGAL, and γH2AX—whereas NAT10 silencing reversed these effects (Fig. 3H, I). Consistently, immunofluorescence staining revealed reduced p16 and γH2AX signals in NAT10-deficient HK-2 cells compared with high-glucose–treated controls (Fig. 3J, K). Together, these findings demonstrate that NAT10 knockdown suppresses PAPP-A upregulation and attenuates high-glucose–induced tubular epithelial cell senescence and injury.

### NAT10 promotion of renal tubular epithelial cell senescence and injury through the PAPP-A/p53 pathway

**Fig. 4:**
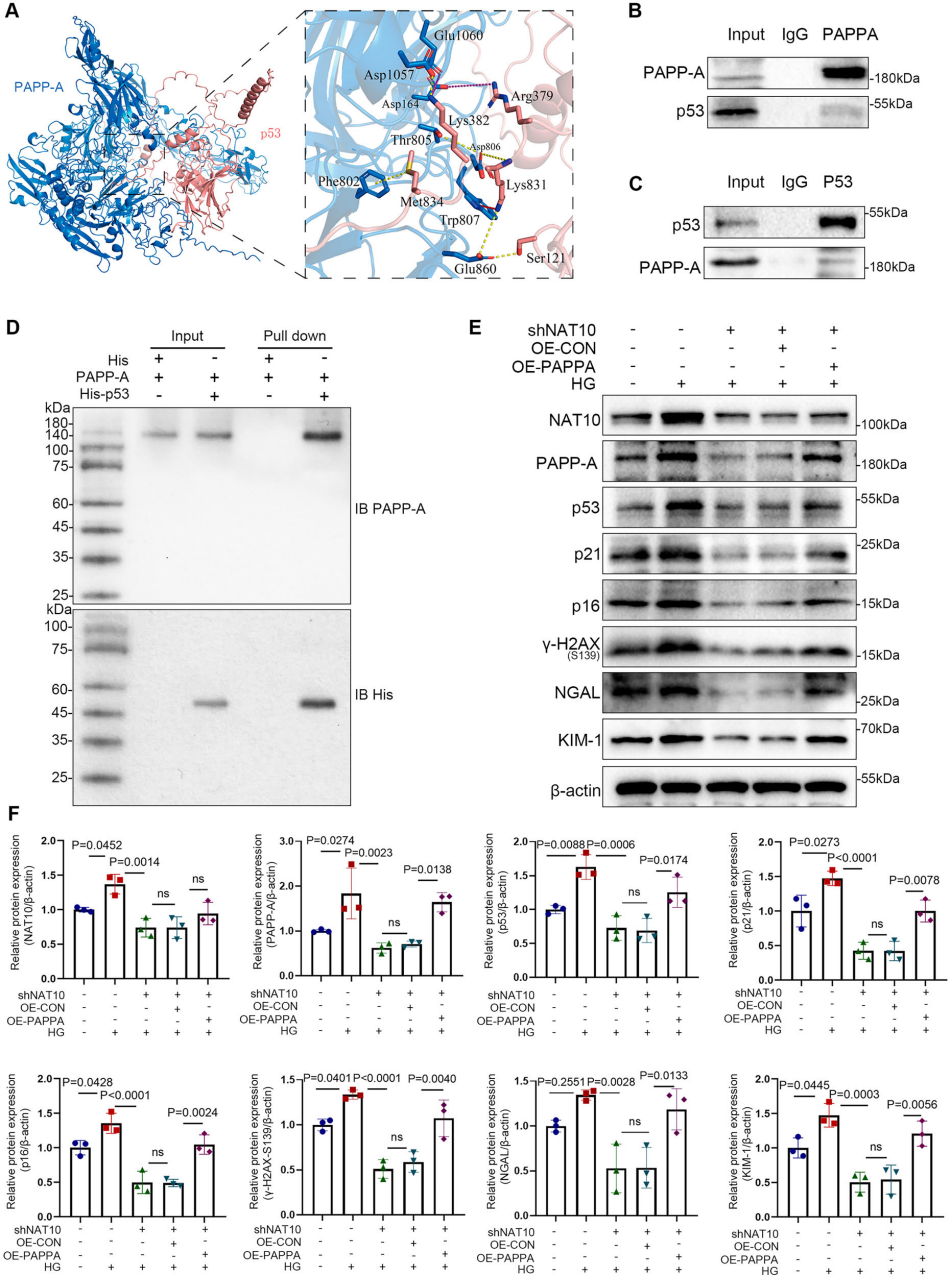
PAPP-A is involved in the regulation of NAT10 in diabetic nephropathy. **A** Molecular docking model of PAPP-A and p53. The yellow dotted lines represent hydrogen bonds, the purple dotted lines represent electrostatic interactions, and the red dotted lines represent cation-pi interactions. **B**, **C** The interaction between PAPP-A and p53 proteins was detected by co-immunoprecipitation. **D** The interaction between PAPP-A and p53 proteins was detected by His-pull down. **E** Western blot was used to detect NAT10, PAPP-A, NGAL, KIM-1, p53, p21, p16, and γH2AX(S139) expression in different groups (*n* = 3). The stimulation time of shNAT10 and OE-PAPP-A was 48 h. The stimulation condition of glucose was 30 mM/48 h. **F** Densitometric analyses of the expression of NAT10, PAPP-A, NGAL, KIM-1, p53, p21, p16, γH2AX, presented as relative levels normalized to β-actin levels.

To elucidate how PAPP-A regulates p53 and cellular senescence, we first analyzed the three-dimensional protein structures of PAPP-A and p53 predicted by AlphaFold3. The predicted template modeling (pTM) score was −0.61, indicating that the overall fold of the complex closely resembles the native structure. Structural visualization using PyMOL revealed multiple interacting amino-acid residues at the protein–protein interface (Fig. 4A), suggesting that PAPP-A directly interacts with p53 to mediate downstream regulatory functions. The interaction between PAPP-A and p53 was demonstrated by co-immunoprecipitation (Co-IP) and His pull-down assays. In the Co-IP assays, total protein lysates were immunoprecipitated with anti-PAPP-A or anti-p53 antibodies, and reciprocal co-precipitation of PAPP-A and p53 was detected (Fig. 4B, C). In addition, the His pull-down assay further confirmed a direct interaction between PAPP-A and p53 (Fig. 4D).

To further investigate the regulatory relationship between NAT10 and PAPP-A, a PAPP-A-overexpressing plasmid was constructed. Under high-glucose conditions, the elevated expression of PAPP-A, p53, p21, p16, γH2AX (Ser139), KIM-1, and NGAL in HK-2 cells was attenuated by NAT10 inhibition. Moreover, PAPP-A overexpression partially reversed the protective effects conferred by NAT10 inhibition (Fig. 4E, F).

### Renal tubule-specific knockout of NAT10 attenuates renal tubular epithelial cell senescence in STZ-induced T1DM mouse models

**Fig. 5:**
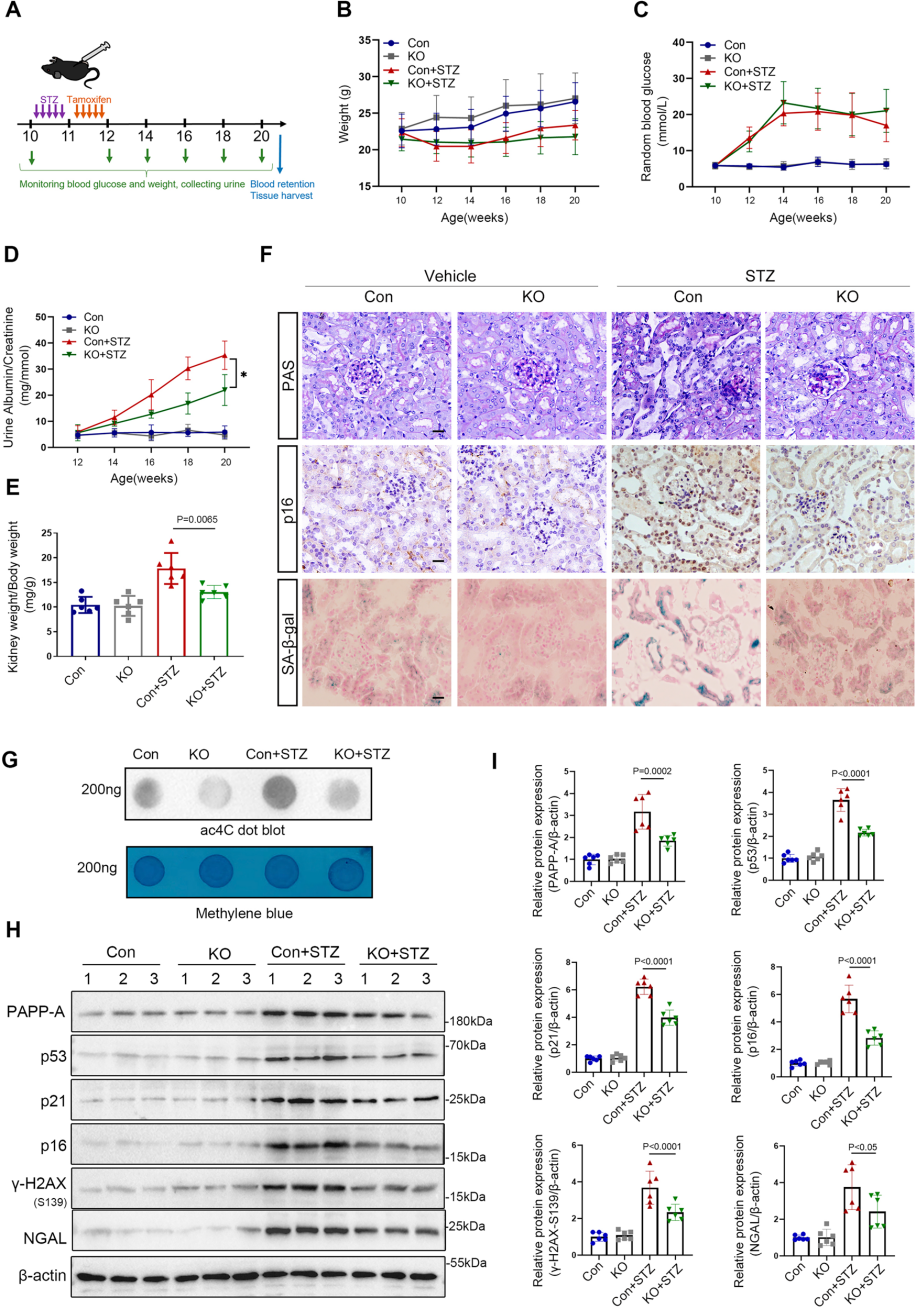
Renal tubule-specific deletion of NAT10 ameliorates kidney in STZ-injured mice. **A** Experimental design. STZ were injected into mice at the 10th weeks. Blood glucose levels and body weights were monitored every two weeks from the 10th to the 20nd week. Renal tissues were collected at the 20nd week. **B** Body weights of each group at different time points. **C** Random blood glucose levels of each group at different time points. **D** Quantification of urine albumin levels adjusted by urine creatinine concentrations. *P < 0.05 (*n* = 6). **E** Kidney to body weight ratios were measured at the endpoint (*n* = 6). **F** Representative PAS staining (Scale bar, 20 μm) shows renal tubular injury in different groups. Immunohistochemical detection of P16 expression, along with representative SA-β-gal staining (Tissue sections were counterstained with nuclear fast red after SA-β-Gal staining to clearly display the morphology of renal tubules), demonstrates renal tubular aging in different groups. The scale bars = 20 μm. **G** The ac4C level of the kidney in different groups of mice were determined using ac4C dot blot assay. **H** Western blot was used to detect PAPP-A, p53, p21, p16, γH2AX, and NGAL expression in different groups (*n* = 6). **I** Densitometric analysis of PAPP-A, p53, p21, p16, γH2AX, and NGAL. The relative intensities of the bands were normalized to the intensities of the respective β-actin signal.

To investigate whether NAT10 regulates PAPP-A within renal tubular epithelial cells in type 1 diabetes, we generated mice with tubule-specific deletion of NAT10 using a tamoxifen-inducible Cre–loxP system (Fig. S3A). Efficient recombination was confirmed by RT–PCR and immunoblotting (Fig. S3B, C). Immunofluorescence staining further verified the lack of NAT10 signaling in the renal tubules of Cdh16-Cre/NAT10^flox/flox^ mice, whereas it was detected in control mouse kidneys (Fig. S3D). Under physiological conditions, cKO mice were viable and exhibited normal development, behavior, weight (Fig. 5B), blood glucose (Fig. 5C), urinary albumin excretion (Fig. 5D), kidney-to-body weight ratio (Fig. 5E), serum creatinine (Fig. S3E) and kidney histology (Fig. 5F) compared with their control littermates. We subsequently induced type 1 diabetes in both cKO and control mice using STZ following intraperitoneal administration of tamoxifen, and the animals were sacrificed 20 weeks later. As shown in Fig. 5D, E, *NAT10* knockout reduced the urine albumin-to-creatinine ratio and the kidney-to-body weight ratio in STZ-treated diabetic mice. Periodic acid–Schiff (PAS) staining and SA-β-gal staining demonstrated that NAT10 cKO attenuated STZ-induced tubular epithelial injury and renal tubular epithelial cell senescence (Fig. 5F). ac4C dot blot analyses revealed a further decline in ac4C acetylation levels in kidneys of STZ-treated cKO mice compared with diabetic controls (Fig. 5G). Western blot analysis revealed that the expression of PAPP-A and canonical senescence-associated markers—including p53, p21, p16, γH2AX (S139), and NGAL—was substantially elevated in diabetic kidneys, whereas *NAT10* conditional knockout reversed these effects (Fig. 5H, I). Collectively, these results show that renal tubule-specific deletion of NAT10 ameliorates proteinuria and renal tubular epithelial cell senescence in type 1 diabetes mouse models.

### NAT10 knockdown attenuates renal tubular epithelial cell injury and renal tubular epithelial cell senescence in db/db mouse models of T2DM

**Fig. 6:**
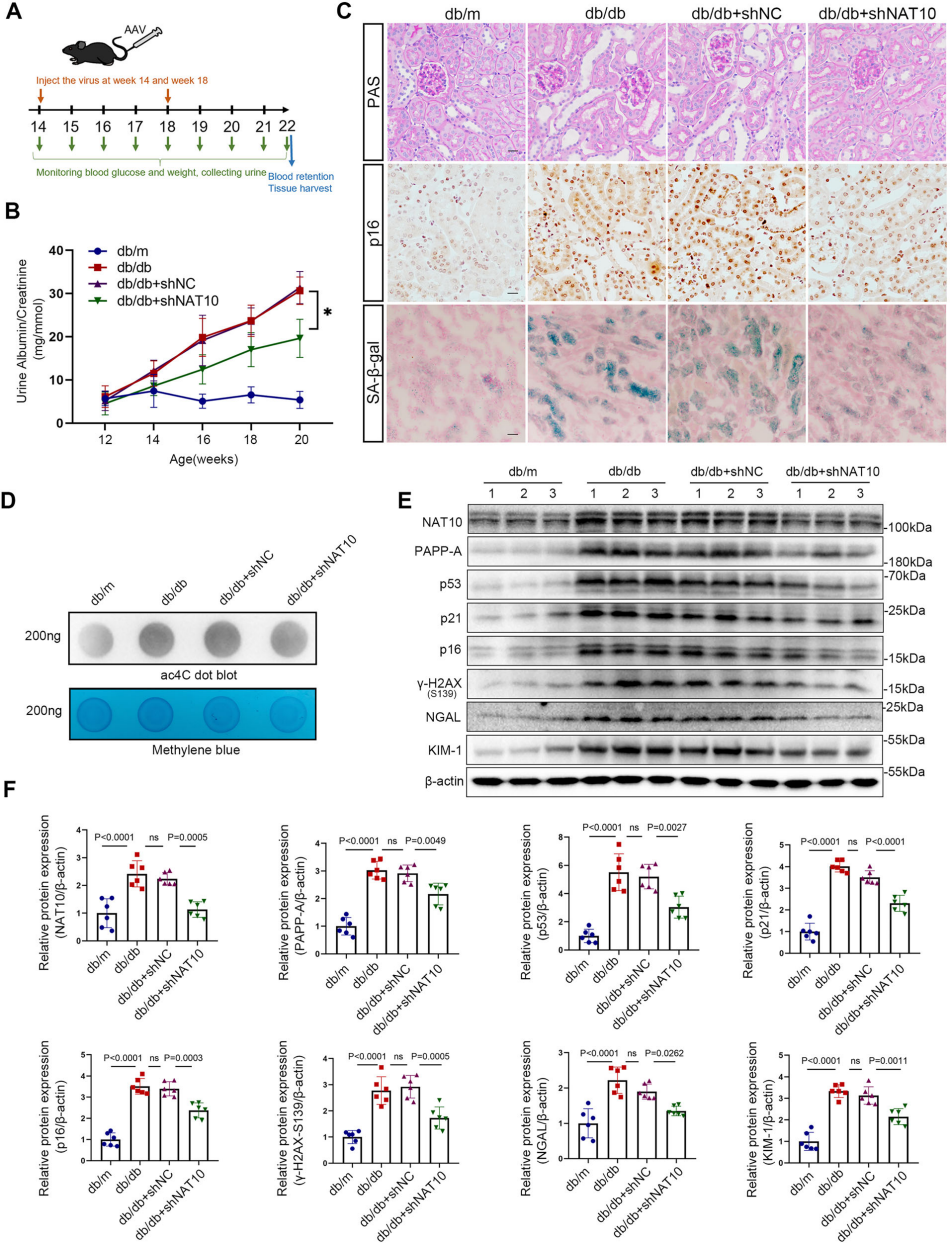
NAT10 knockdown attenuates renal tubular epithelial cell senescence in db/db mice. **A** Experimental design. Viruses were injected into mice at the 14th and 18th weeks. Blood glucose levels and body weights were monitored every two weeks from the 14th to the 22nd week. Renal tissues were collected at the 22nd week. **B** Quantification of urine albumin levels adjusted by urine creatinine concentrations. *P < 0.05 (*n* = 6). **C** Representative PAS staining (Scale bar, 20 μm) shows renal tubular injury in different groups. Immunohistochemical detection of p53, p21, and p16 expression, along with representative SA-β-gal staining, demonstrates renal tubular aging in different groups. The scale bars = 20 μm. **D** The ac4C level of the kidney in different groups of mice was determined using ac4C dot blot assay. **E** Western blot was used to detect NAT10, PAPP-A, p53, p21, p16, γH2AX, NGAL and KIM-1 expression in different groups (*n* = 6). **F** Densitometric analysis of NAT10, PAPP-A, p53, p21, p16, γH2AX, NGAL and KIM-1. The relative intensities of the bands were normalized to the intensities of the respective β-actin signal.

To determine whether NAT10 regulates PAPP-A in renal tubular epithelial cells of T2DM mice, we constructed an AAV vector designed for renal tubule-specific knockdown of NAT10. The detailed injection protocol is illustrated in Fig. 6A. Administration of either the control AAV or shNAT10 virus had no significant effect on body weight or blood glucose levels (Fig. S4A, B). The shNAT10 virus effectively reduced NAT10 expression. As shown in Fig. 6B and S4C, NAT10 knockdown decreased urine albumin-to-creatinine ratios and serum creatinine. Histopathological staining revealed that the renal tubules of mice exhibited dilation, epithelial cell shedding, and brush-border disruption in db/db mice, all of which were alleviated by shNAT10 treatment (Fig. 6C). Immunohistochemical staining confirmed that shNAT10 injection decreased p16 expression in renal tubules. Additionally, SA-β-gal staining further demonstrated that NAT10 knockdown alleviated kidney aging (Fig. 6C). Dot blot assays showed that renal ac4C levels were reduced in db/db mice receiving shNAT10 compared with those treated with control virus (Fig. 6D). Western blot analysis revealed that expression of NAT10, PAPP-A, and key senescence markers—including p53, p21, p16, and γH2AX (S139)—was substantially lower in shNAT10-treated db/db mice. Moreover, the tubular injury markers NGAL and KIM-1, which were markedly elevated in db/db mice, were also reduced following NAT10 knockdown (Fig. 6E, F). Collectively, these results demonstrate that renal tubule-specific knockdown of NAT10 ameliorates renal tubular aging and injury in type 2 diabetic mouse models.

### Pharmacologic targeting of NAT10 attenuates renal tubular epithelial cell injury and renal tubular epithelial cell senescence in experimental DN

**Fig. 7:**
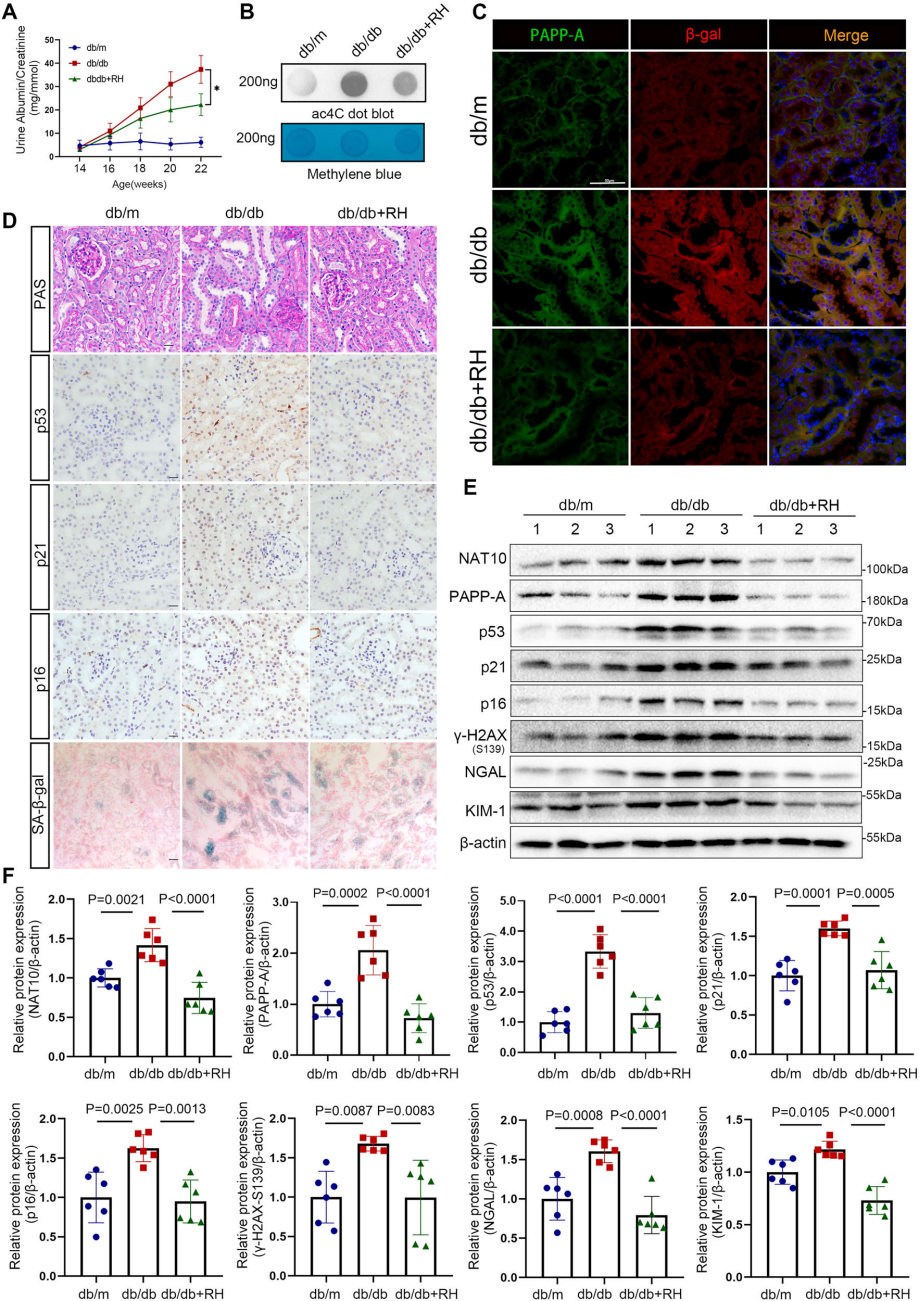
Remodelin therapy attenuates renal tubular epithelial cell senescence in vivo. **A** Quantification of urine albumin levels adjusted by urine creatinine concentrations. *P < 0.05 (*n* = 6). **B** The ac4C level of kidney in different groups mice were determined using ac4C dot blot assay. **C** Representative micrographs show the expression of PAPP-A and β-gal in different groups. Green represents PAPP-A, Red represents β-gal and blue represents DAPI. The scale bars = 50 μm. **D** Representative PAS staining (Scale bar, 20 μm) shows renal tubular injury in different groups. Immunohistochemical detection of P53, P21, and P16 expression, along with representative SA-β-gal staining, demonstrates renal tubular aging in different groups. The scale bars = 20 μm. **E** Western blot was used to detect NAT10, PAPP-A, p53, p21, p16, γH2AX, NGAL and KIM-1 expression in different groups (*n* = 6). **F** Densitometric analysis of NAT10, PAPP-A, p53, p21, p16, γH2AX, NGAL and KIM-1. The relative intensities of the bands were normalized to the intensities of the respective β-actin signal.

Extensive preclinical evidence indicates that NAT10 is a druggable target across multiple organ systems, with small-molecule inhibitors such as Remodelin [25]. To assess whether systemic administration of Remodelin regulates NAT10 in renal tubular epithelial cells and influences renal tubular epithelial cell senescence in vivo, we administered Remodelin at a dose of 5 mg/kg to murine models of DN. The specific treatment protocol is illustrated in Fig. S5A. After calculating the body weight and blood glucose levels, it was found that Remodelin had no significant impact on either parameter (Fig. S5B, C). Remodelin therapy markedly attenuated urine albumin-to-creatinine ratios and serum creatinine in db/db mice (Figs. 7A and S5D). Histopathological staining revealed broken brush borders, tubular dilation, and epithelial cell loss in db/db mice, which were alleviated by Remodelin treatment (Fig. 7D). Remodelin therapy successfully blocked NAT10 in renal tubules in vivo, which was accompanied by decreased expression of PAPP-A and KIM-1 and attenuation of renal tubular epithelial cell senescence (Figs. 7D and S5E, F). In addition, we performed immunofluorescence staining to examine the expression of PAPP-A and β-gal in kidney tissues (Fig. 7C). The results showed that the expression of both proteins was markedly decreased after Remodelin treatment, which was consistent with the WB findings and further supported that Remodelin alleviates renal tubular epithelial cell senescence. The ac4C dot blot revealed that ac4C acetylation was reduced in the kidneys of db/db mice injected with Remodelin compared with those receiving the vehicle (Fig. 7B). WB analysis further showed that Remodelin diminished expression of canonical senescence markers—including p53, p21, p16, and γH2AX (S139)—as well as renal injury markers NGAL and KIM-1 (Fig. 7E, F). Collectively, these results establish that systemic inhibition of NAT10 with Remodelin effectively reduces tubular injury and ameliorates renal tubular epithelial cell senescence in vivo.

**Fig. 8:**
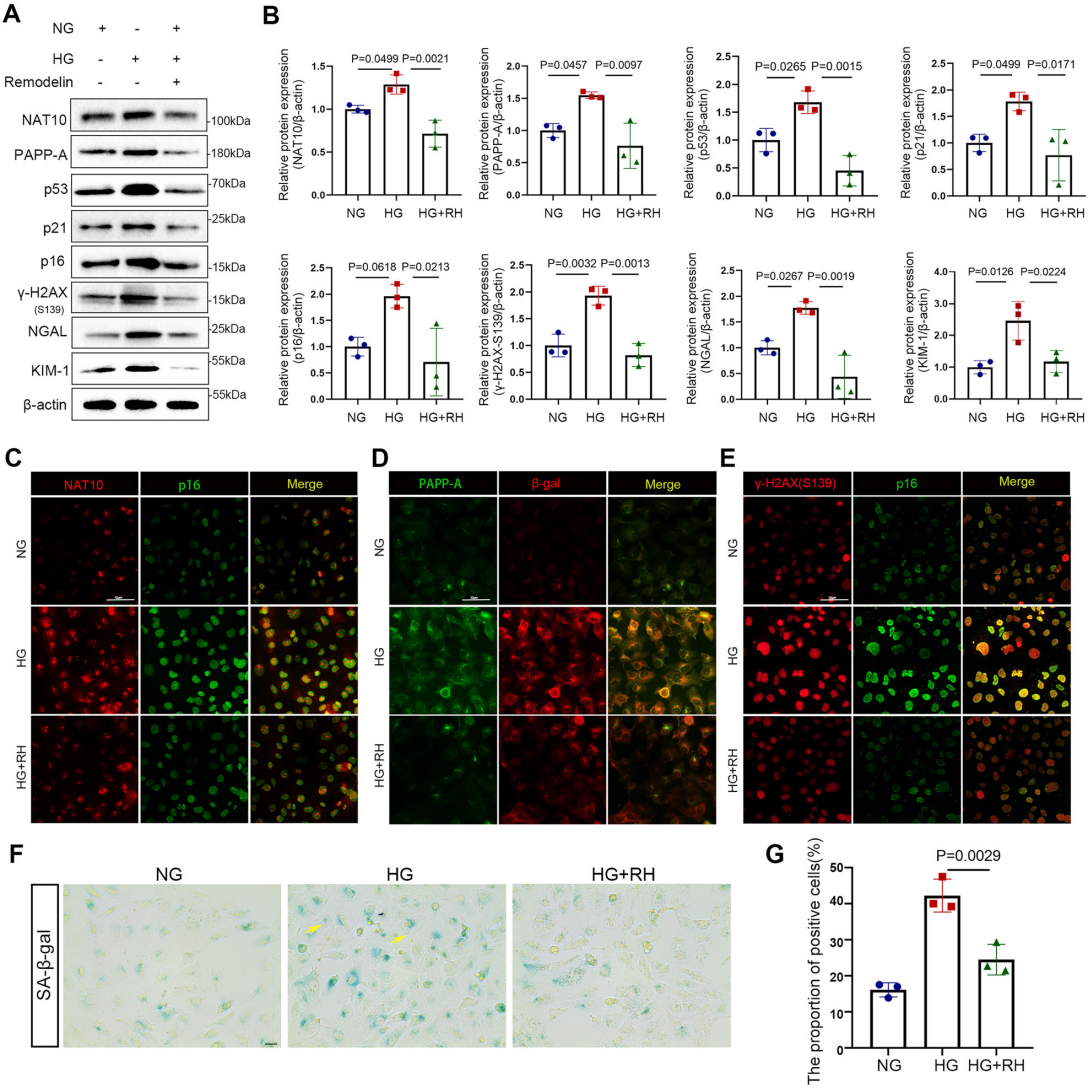
Remodelin therapy attenuates renal tubular epithelial cell senescence in vitro. **A** Western blot was used to detect NAT10, PAPP-A, p53, p21, p16, γH2AX, NGAL and KIM-1 expression in different groups (*n* = 3). The stimulation condition of Remodlin was 20 mM/24 h. The stimulation condition of glucose was 30 mM/48 h. **B** Densitometric analysis of NAT10, PAPP-A, p53, p21, p16, γH2AX, NGAL and KIM-1. The relative intensities of the bands were normalized to the intensities of the respective β-actin signal. **C** Representative micrographs show the expression of NAT10 and p16 in different groups. Green represents p16, and Red represents NAT10. The scale bars = 50 μm. **D** Representative micrographs show the expression of PAPP-A and β-gal in different groups. Green represents PAPP-A, and Red represents β-gal. The scale bars = 50 μm. **E** Representative micrographs show the expression of p16 and γH2AX(S139) in different groups. Green represents p16, and Red represents γH2AX(S139). The scale bars = 50 μm. **F** Representative micrographs showing SA-β-gal staining in different groups. The scale bars = 20 μm. **G** The quantification of the SA-β-gal-positive cells was determined after three biological replicates.

We further validated the effects of Remodelin in HK-2 cells and obtained results consistent with the in vivo experiments described above. Remodelin treatment successfully blocked NAT10 in renal tubules in vitro (Figs. 8A and S6A). WB analysis revealed that Remodelin reversed the high-glucose-induced upregulation of PAPP-A, p53, p21, p16, γH2AX (S139), NGAL, and KIM-1 (Fig. 8A, B). Immunofluorescence staining further confirmed the reduction of NAT10, p16, γH2AX (S139), PAPP-A, β-Gal and KIM-1 in Remodelin-treated cells (Figs. 8C–E and S6B). In addition, SA-β-Gal staining revealed trends consistent with those observed in WB and immunofluorescence analyses (Fig. 8F). Furthermore, we conducted the aforementioned experiments in primary renal tubular epithelial cells and obtained results consistent with those observed in HK2 cells (Fig. S7). These findings suggest that Remodelin suppresses NAT10 expression in renal tubular epithelial cells, thereby mitigating cell senescence and injury in vitro.

## Discussion

ac4C is a widespread and reversible RNA modification in mammalian cells that enhances translation efficiency and mRNA stability [26, 27]. Recent evidence points to abnormal ac4C modifications are associated with numerous human diseases, including acute myeloid leukaemia [28], cardiac dysfunction [29], ischemic stroke [25], liver fibrosis [30], and various cancers [31–33]. However, the function and mechanism of RNA ac4C modification in DN remain poorly understood. NAT10 is currently the only known ac4C “writer” enzyme, catalyzing acetylation not only on mRNA but also on rRNA, tRNA, and long non-coding RNAs [14, 26, 34]. This research highlights the critical role of NAT10 in renal tubular epithelial cell senescence and injury. Our earlier study illustrated the function of NAT10-mediated senescence in adriamycin nephropathy [35], however, this is the first study to elucidate the significance of NAT10 in senescence in DN. In this study, we analyzed the single-cell databases. By analysis of Wilson et al. [23], which examined patients with early-stage human DN, we found NAT10 increased in renal tubules in human samples. Our data also confirmed that NAT10 was highly induced in renal tubules in both the db/db and the STZ-induced DN models, as well as in human kidney and high glucose-stimulated HK-2 cells. Importantly, we demonstrated that increased tubular NAT10 expression correlates with accelerated renal tubular epithelial cell senescence and functional decline in patients with DN.

To investigate the mechanism of NAT10 in DN, ac4C-RIP-seq and RNA-seq were performed in db/db mice. We found that kidneys from db/db mice exhibited increased ac4C modification of *PAPP-A* mRNA. This was further validated by acRIP-PCR, which confirmed the presence of ac4C modification on *PAPP-A* mRNA. In addition, we confirmed that NAT10 directly binds to *PAPP-A* mRNA. Based on these results, we concluded that changes in PAPP-A expression were attributable to NAT10-mediated ac4C modification of its mRNA. Previous studies have demonstrated that NAT10 enhances mRNA translation efficiency and stability through ac4C modification [14, 36], thereby contributing to diverse cellular processes. To assess *PAPP-A* mRNA stability after NAT10 knockdown, we performed an actinomycin D assay, which revealed reduced stability. Collectively, these findings indicate that NAT10 promotes biological processes by enhancing *PAPP-A* mRNA stability via ac4C modification.

PAPP-A, a metalloproteinase, is critical for regulating the bioavailability of insulin-like growth factor (IGF) within tissues [37]. Accumulating evidence implicates PAPP-A in cellular senescence [38], and genetic studies show that homozygous deletion of PAPP-A markedly extends lifespan in mice [39]. Moreover, Jing et al. reported that PAPP-A activation mediates aging induced by sirtuin deficiency [40], highlighting its close association with aging. Elevated PAPP-A expression has also been observed in the glomeruli of patients with DN [41]. A recent study reported that PAPP-A is involved in the pathogenesis and progression of polycystic kidney disease [42]. Despite these observations, the mechanisms underlying PAPP-A upregulation and its contribution to renal tubular aging in DN remain poorly defined. In the present study, we found that PAPP-A expression is significantly increased in renal tubular epithelial cells from patients with DN. We demonstrated that PAPP-A interacts with p53 to promote senescence in renal tubular epithelial cells. Our data suggest that NAT10 knockdown suppresses PAPP-A expression and protects renal tubules by reducing senescence and injury, whereas PAPP-A overexpression reverses this protective effect.

Cellular senescence is associated with numerous diseases, including cardiovascular diseases [43, 44], kidney diseases [45–48], tumors [49], and so on. Recent studies have revealed that tubular epithelial cells in diabetic nephropathy exhibit cellular senescence [50, 51]. Among the key regulators of cellular senescence, p53 has been widely recognized as a central mediator of stress responses and aging-related signaling. As a critical tumor suppressor and transcription factor, p53 promotes cell cycle arrest and senescence primarily through activation of downstream targets such as p21 and other senescence-associated genes, thereby playing a pivotal role in the regulation of cellular aging processes. In kidney injury, activation of the p53 pathway has been frequently linked to tubular epithelial cell senescence and disease progression [35]. How does NAT10 modulate renal tubular epithelial cell senescence? Our data suggest that NAT10 regulates PAPP-A expression and our protein–protein interaction prediction (AlphaFold) together with Co-IP experiments further indicate a potential physical interaction between PAPP-A and p53, which may directly influence senescence signaling by interacting with the key mediator p53. Thus, it is conceivable that NAT10 hyperactivity increases p53 expression, which is required for its activity [52], thereby promoting senescence signaling and renal tubular epithelial cell senescence. Consistent with this hypothesis, TLR2 [35] and TGFβ1 [30] have also been implicated in modulating the senescence signaling pathway. Our findings do not exclude the possibility that other NAT10-regulated pathways contribute to renal tubular epithelial cell senescence.

The therapeutic potential of NAT10 inhibition has been explored in several diseases, including cancer [18], ischemic stroke [25] and myocardial infarction [53]. Our previous research demonstrated that inhibiting NAT10 with Remodelin alleviates podocyte aging in a mouse model of doxorubicin-induced nephropathy [35]; However, to date, no studies have examined whether targeting NAT10 confers similar benefits in DN. In the present study, we demonstrated that inhibiting NAT10, through renal tubule-specific deletion, significantly reduced senescence and alleviated damage in STZ-induced T1DM mouse models. Consistently, we also showed that NAT10 inhibition via a renal tubular-targeted adeno-associated virus ameliorated renal tubular epithelial cell senescence in db/db mouse models of T2DM. Moreover, our results demonstrate that pharmacological inhibition of NAT10 using Remodelin protects against renal tubular epithelial cell senescence both in vivo and in vitro. These beneficial effects were accompanied by reduced renal expression of PAPP-A and p53, as well as decreased ac4C levels.

In summary, genetic and pharmacologic targeting of NAT10 downregulates PAPP-A mRNA and protein expression by diminishing the stability of *PAPP-A* mRNA, exerting anti-senescence and protective effects.

## Supplementary information

Supplemental Figure

Original data

## Data Availability

Data will be made available on request.
