# Supplementary material for: NAT10 inhibition alleviates renal tubular epithelial cell senescence by impeding ac4C acetylation of PAPP-A mRNA in diabetic nephropathy
Source: Cell Death Dis. 2026 Jun 11;17(1):738. doi: 10.1038/s41419-026-08970-0 (PMC13478519; doi:10.1038/s41419-026-08970-0)
Supplement: Supplementary file 1 [file 41419_2026_8970_MOESM1_ESM.pdf]

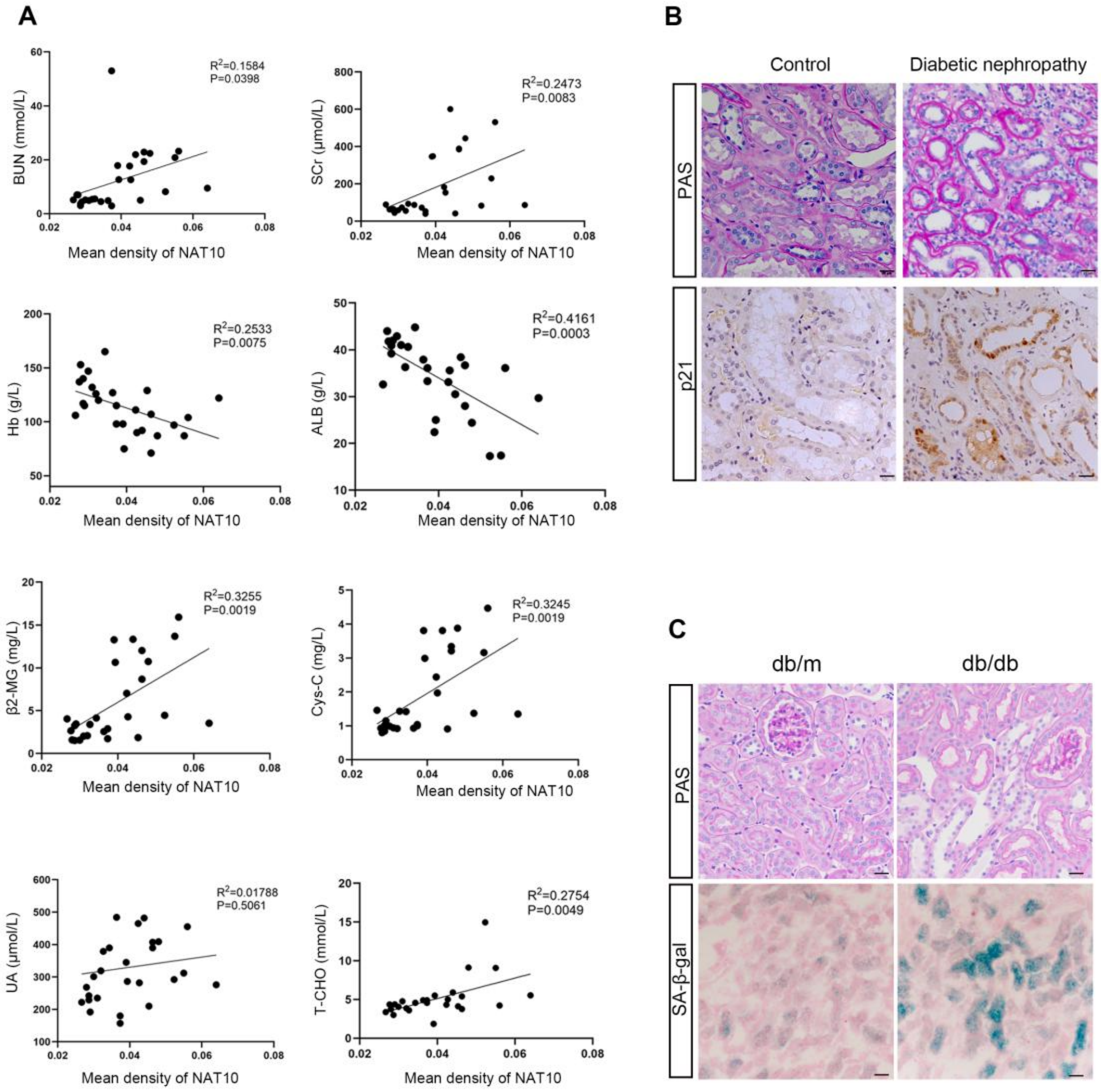

Figure S1. NAT10 expression is associated with clinical indicators in patients with diabetic nephropathy. (A) Correlation analysis between clinical indicators and NAT10. (B) Periodic Acid-Schiff (PAS) staining of human renal tubules. Immunohistochemistry was utilized to detect the expression of P16. The scale bars = 20μm. (C) PAS staining of renal tubules in db/db mice. Representative micrographs showing SA-β-gal staining. The scale bars = 20μm.

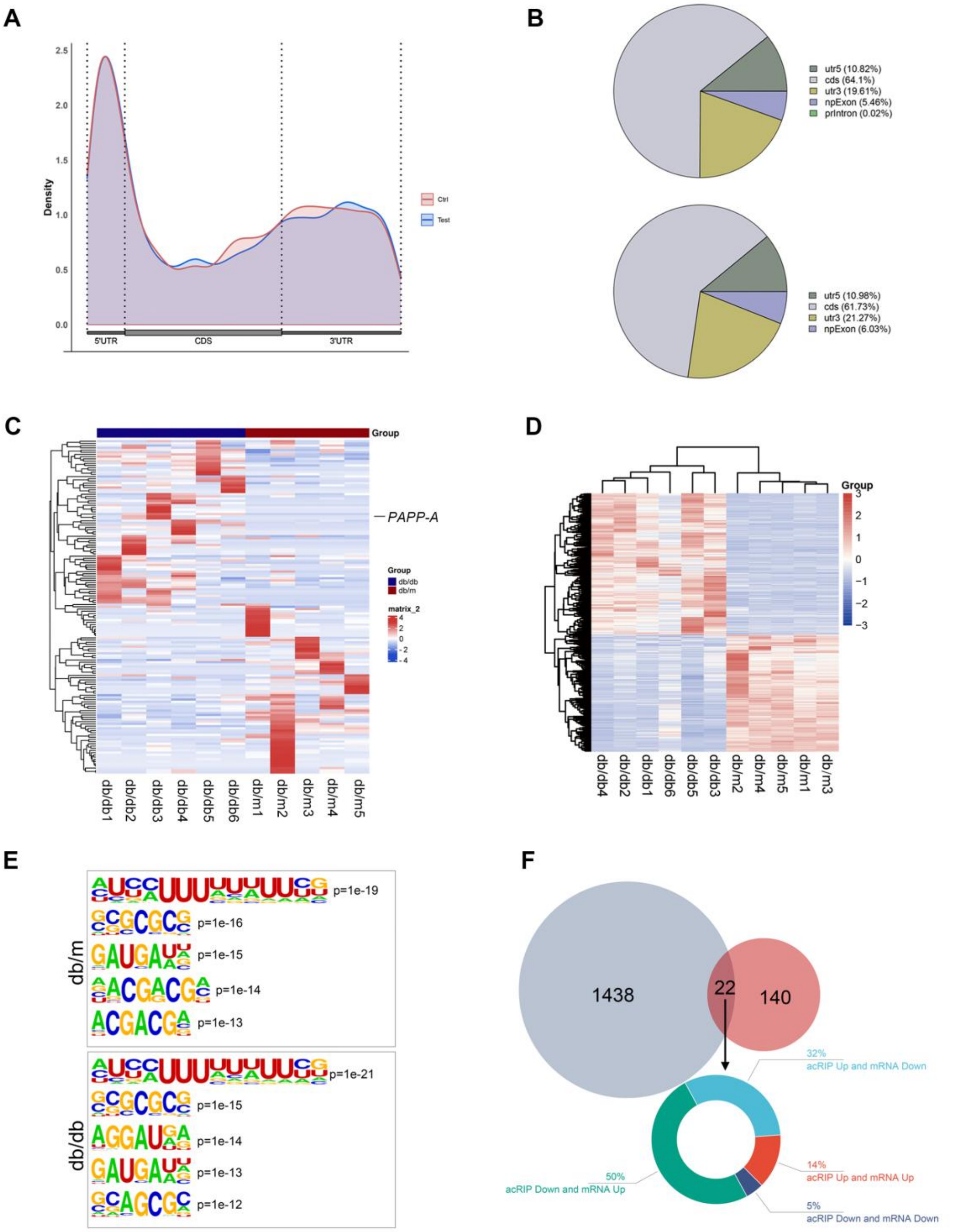

Figure S2. Identification of the profile of ac4C-modified genes in db/db mice. (A) Peak density of the ac4C peaks across the mRNA transcripts. (B) The distribution ratio of ac4C peaks in each transcript region. (C) Heatmap of differentially expressed genes identified by acRIP-seq. (D) Heatmap of differentially expressed genes identified by RNA-seq. (E) Sequence motifs enriched within ac4C peaks were identified by acRIP-seq in db/m mice and db/db mice. (F) Venn diagram of the combined analysis of acRIP-seq and RNA-seq.

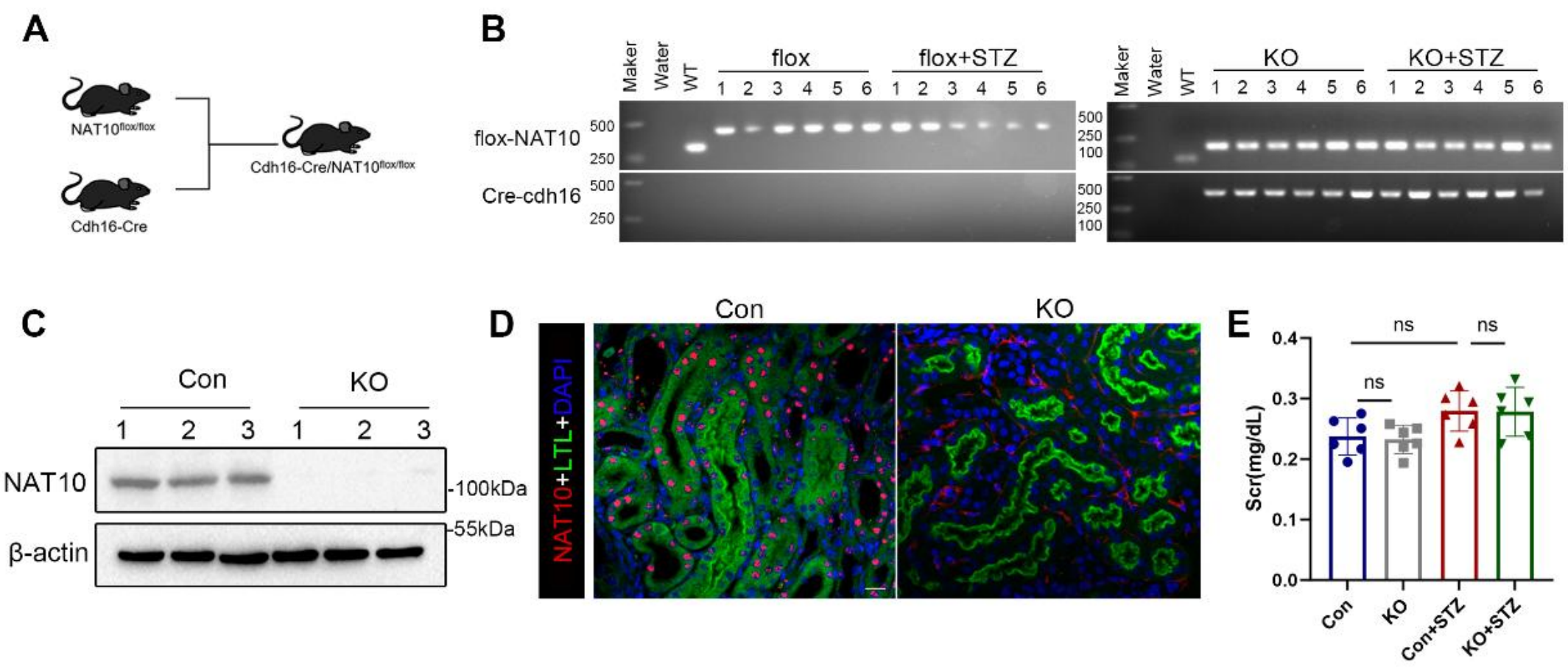

Figure S3. Construction of renal tubular-specific NAT10 deletion mice using Cre/loxP system. (A) Schematic diagram depicts the tamoxifen-inducible tissue specific Cre-loxP gene recombination strategy to target the NAT10 gene selectively in renal tubular cells in adult mice. (B) PCR genotyping of tail DNA from representative mice for detecting the Cre transgenes and the floxed or WT NAT10. All littermates lacking the Cre transgene were designated as control mice. (C) Homogenates of proximal tubules isolated from Con and cKO mouse kidneys were processed for immunoblot analysis for NAT10. β-actin served as a loading control. (D) Kidney specimens from KO or Con mice at 20 weeks old were processed for dual color fluorescence immunohistochemistry staining for NAT10 (red) and LTL (Lotus Tetragonolobus Lectin, green). Sections were counterstained with DAPI (blue). Representative micrographs of laser scanning confocal fluorescence microscopy are shown. Scale bars = 20μm. (E) The levels of serum creatinine in the different groups.

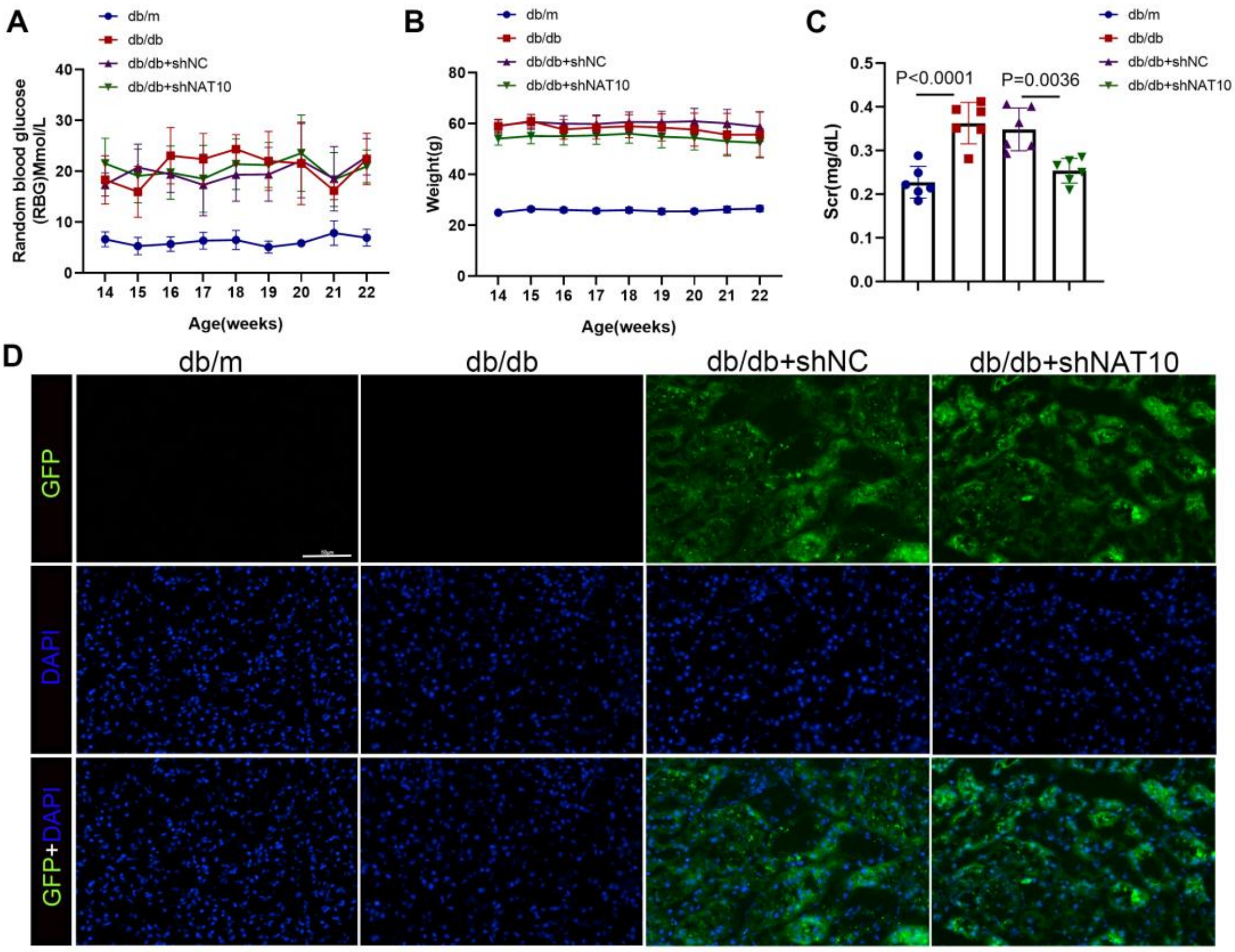

Figure S4. Construction of renal tubular-specific NAT10 knockdown mice using adeno-associated virus (AAV) vector. (A) Body weights of each group at different time points. (B)Random blood glucose levels of each group at different time points. (C) The levels of serum creatinine in the different groups. (D) Micrographs showing GFP fluorescence expression in different groups. Scale bars = 20μm.

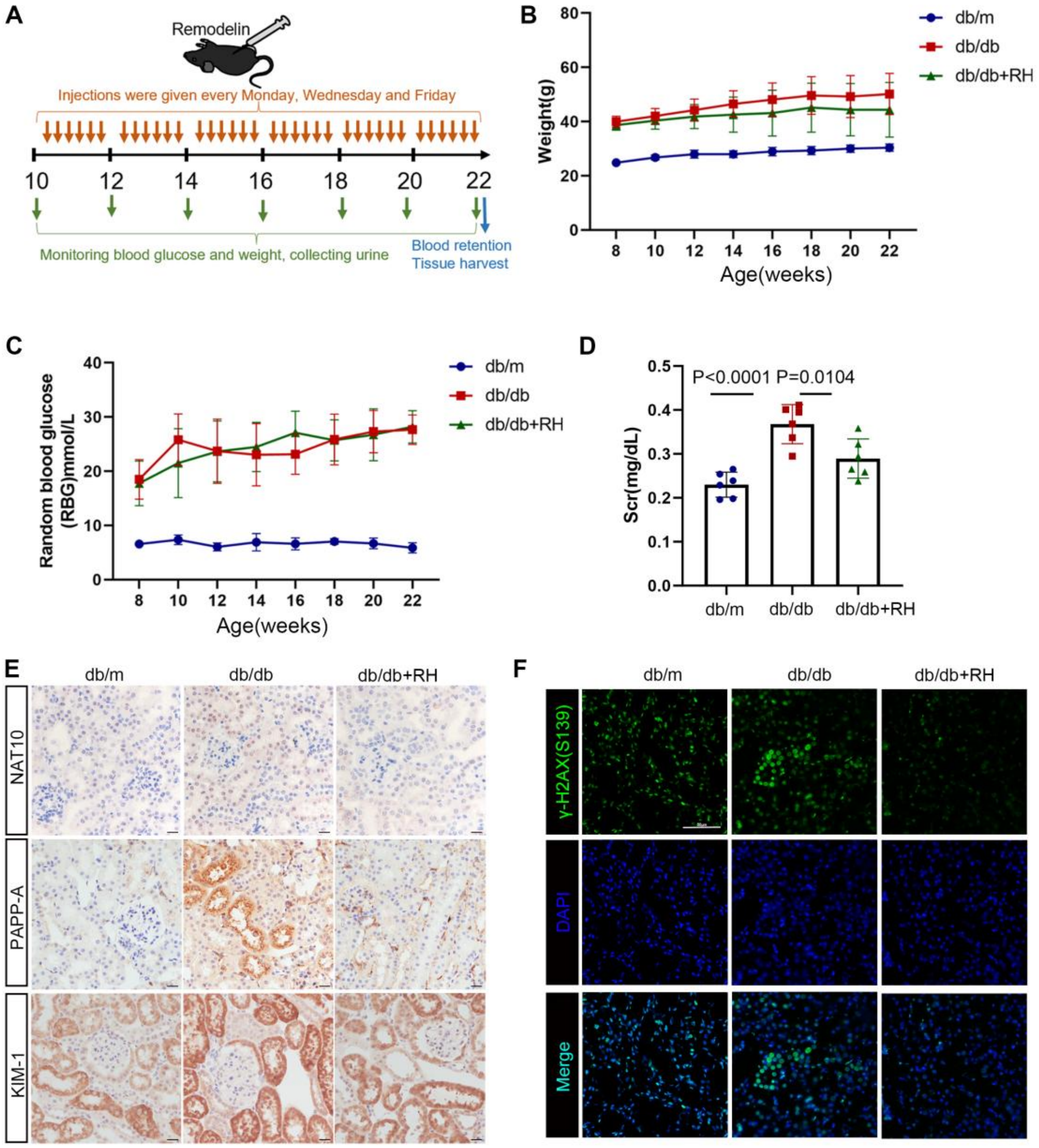

Figure S5. Remodelin therapy attenuates renal tubular damage in vivo. (A) Experimental design. Remodelin was injected intravenously five days a week from 10 weeks to 22 weeks. Stimulation concentration is 5mg/kg. Urine, weight, and blood glucose were collected two weeks at a time. And in 22 weeks collection of the kidney tissue. (B) Body weight at various times. (C) Random blood glucose at various times. (D) The levels of serum creatinine in the different groups. (E) Immunohistochemical detection of NAT10, PAPP-A, and KIM-1 expression. The scale bars=20μm. (F) Representative micrographs show the expression of γH2AX (S139) in different groups. Green represents γH2AX (S139) and blue represents DAPI. The scale bars = 50μm.

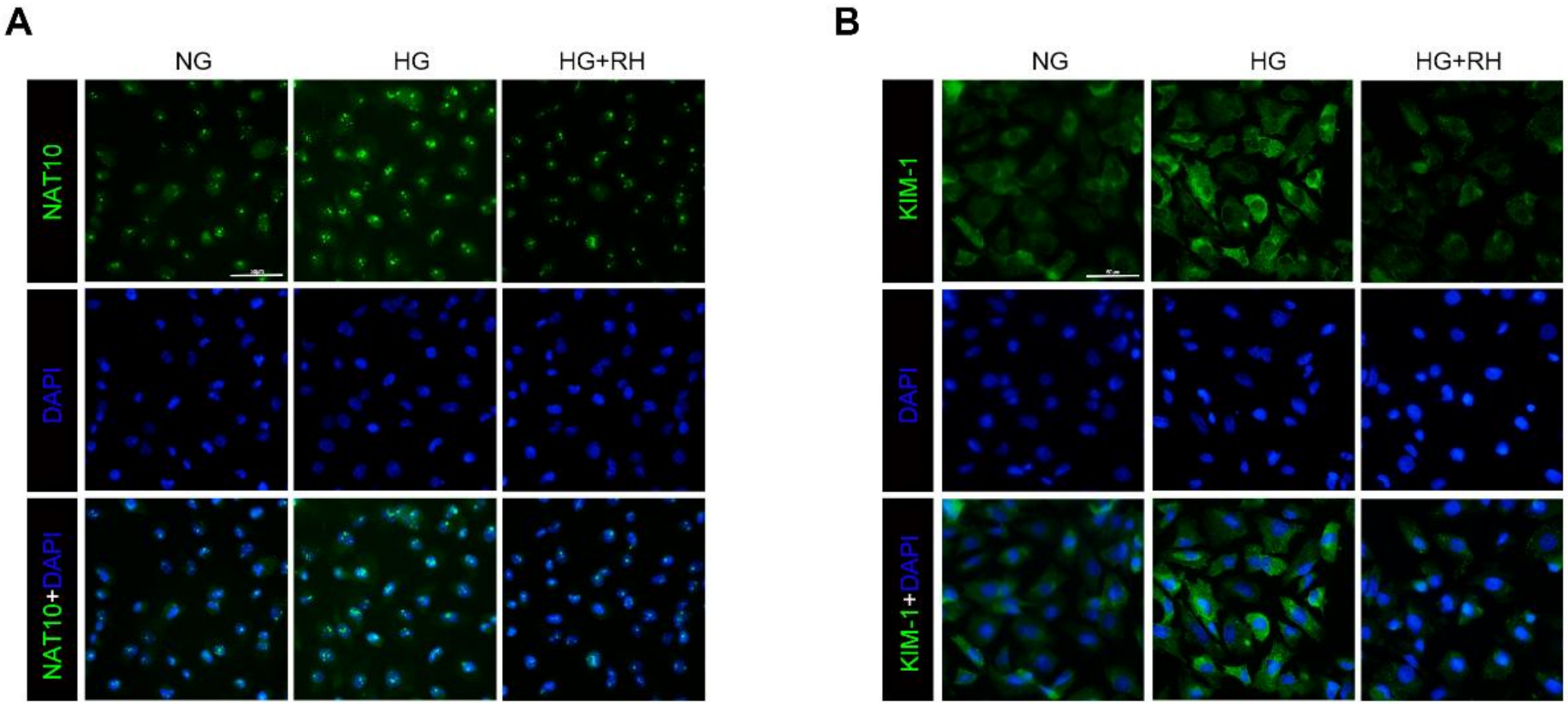

Figure S6. Remodelin therapy attenuates renal tubular aging in vitro. (A) Representative micrographs show the expression of NAT10 in different groups. Green represents NAT10 and blue represents DAPI. The scale bars=50μm. (B) Representative micrographs show the expression of KIM-1 in different groups. Green represents KIM-1 and blue represents DAPI. The scale bars=50μm.

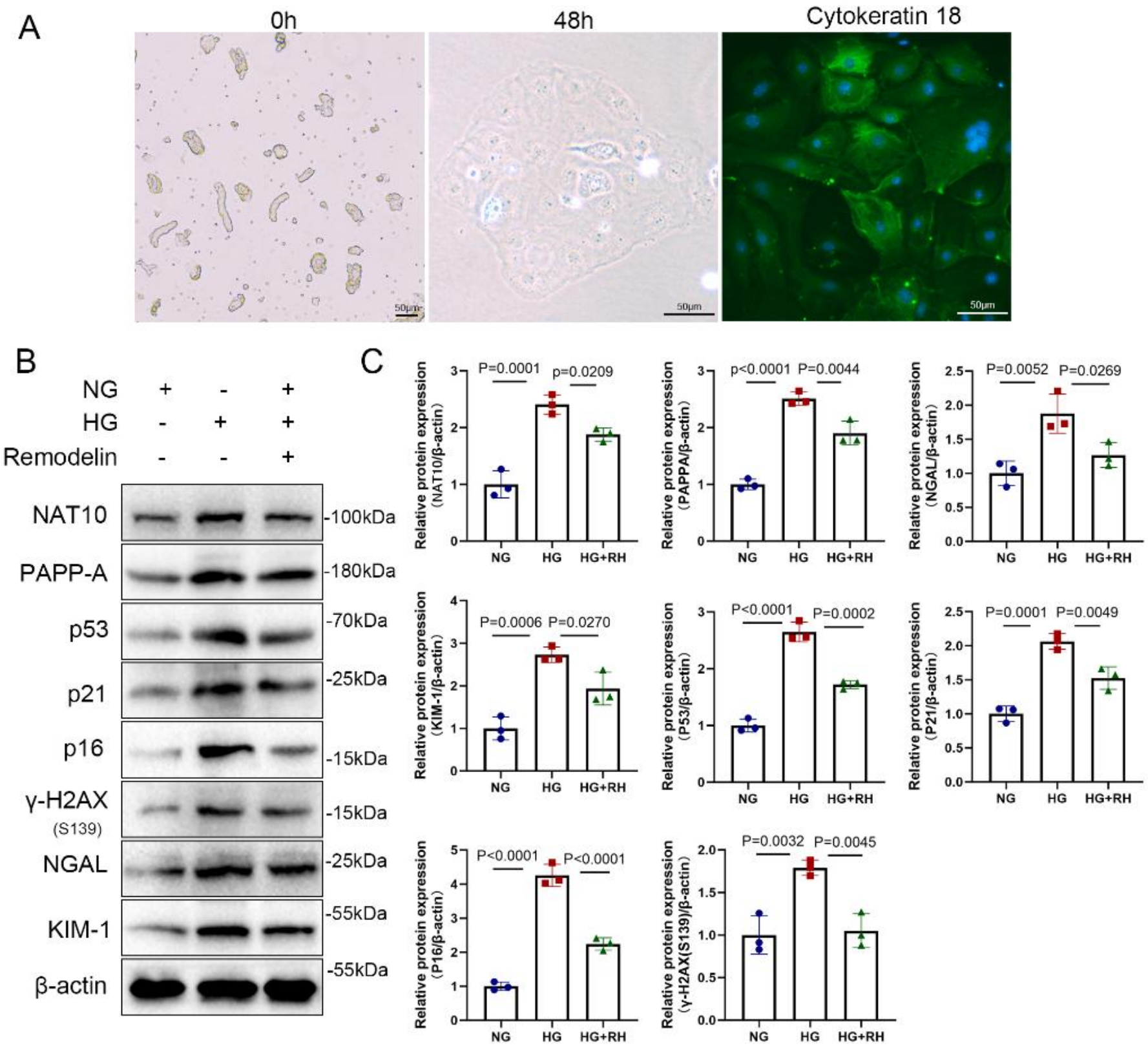

Figure S7. Remodelin therapy attenuates aging in primary renal tubular epithelial cells. (A) Identification of Primary Renal Tubular Epithelial Cells. Green represents Cytokeratin-18 and blue represents DAPI. The scale bars=50μm. (B) Western blot was used to detect NAT10, PAPP-A, p53, p21, p16, γH2AX, NGAL and KIM-1 expression in different groups (n=3). The stimulation condition of Remodlin was 20mM/24h. The stimulation condition of glucose was 30mM/48h. (C) Densitometric analysis of NAT10, PAPP-A, p53, p21, p16, γH2AX, NGAL and KIM-1. The relative intensities of the bands were normalized to the intensities of the respective β-actin signal.
