# Supplementary material for: NAT10 inhibition alleviates renal tubular epithelial cell senescence by impeding ac4C acetylation of PAPP-A mRNA in diabetic nephropathy
Source: Cell Death Dis. 2026 Jun 11;17(1):738. doi: 10.1038/s41419-026-08970-0 (PMC13478519; doi:10.1038/s41419-026-08970-0)
Supplement: Supplementary file 2 [file 41419_2026_8970_MOESM2_ESM.pdf]

Figure 1I

Figure 2E

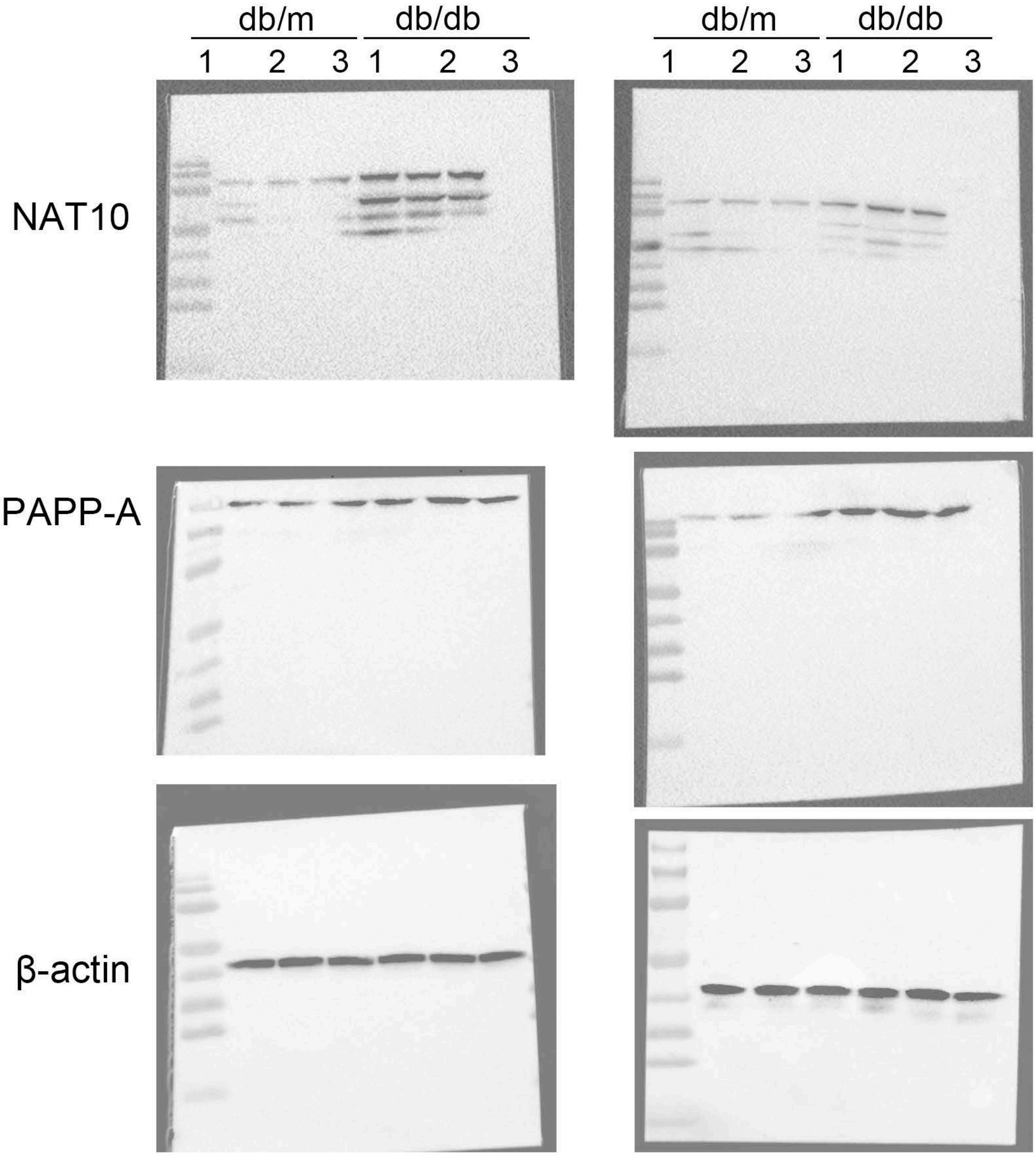

Figure 1L

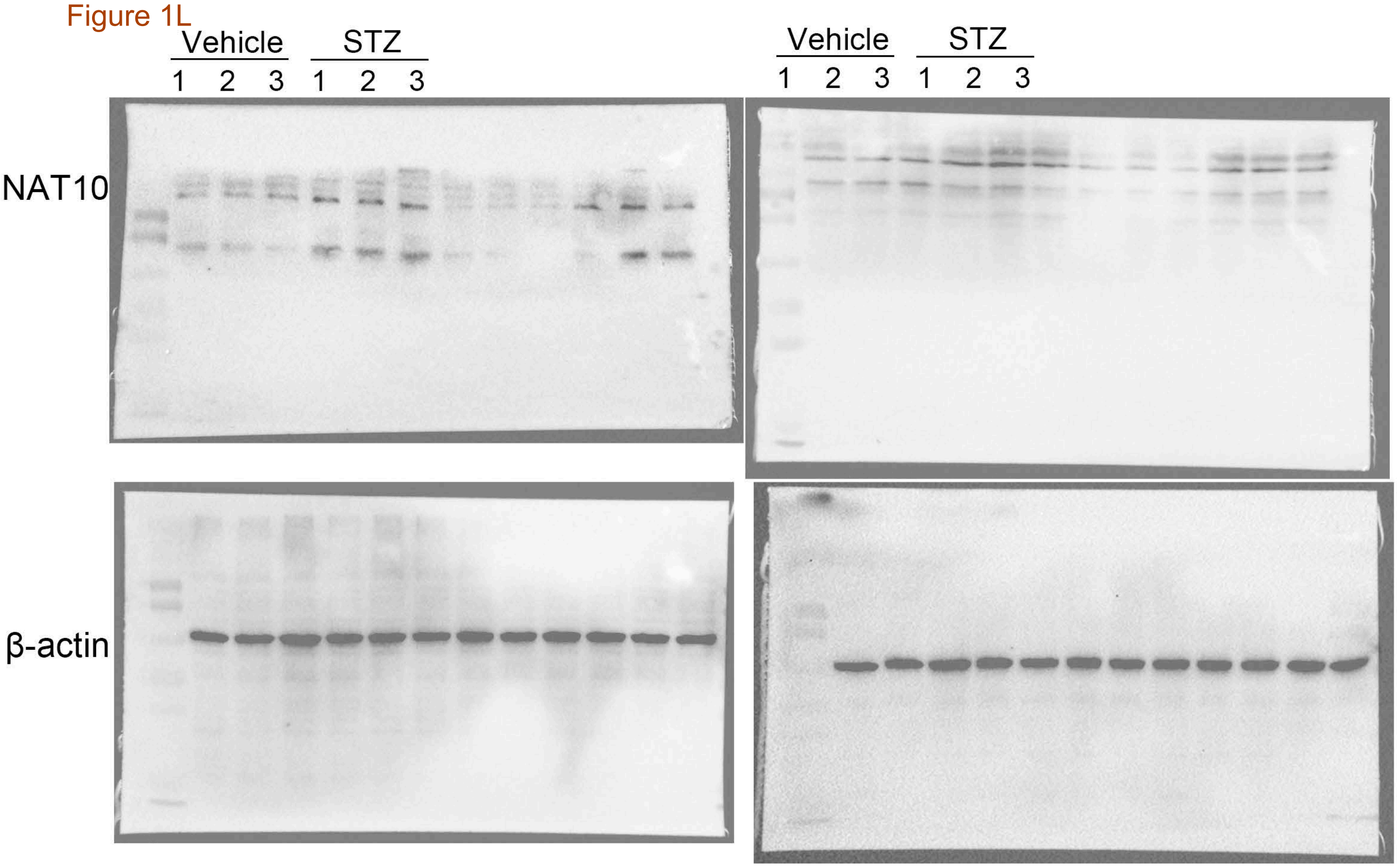

Figure 3A

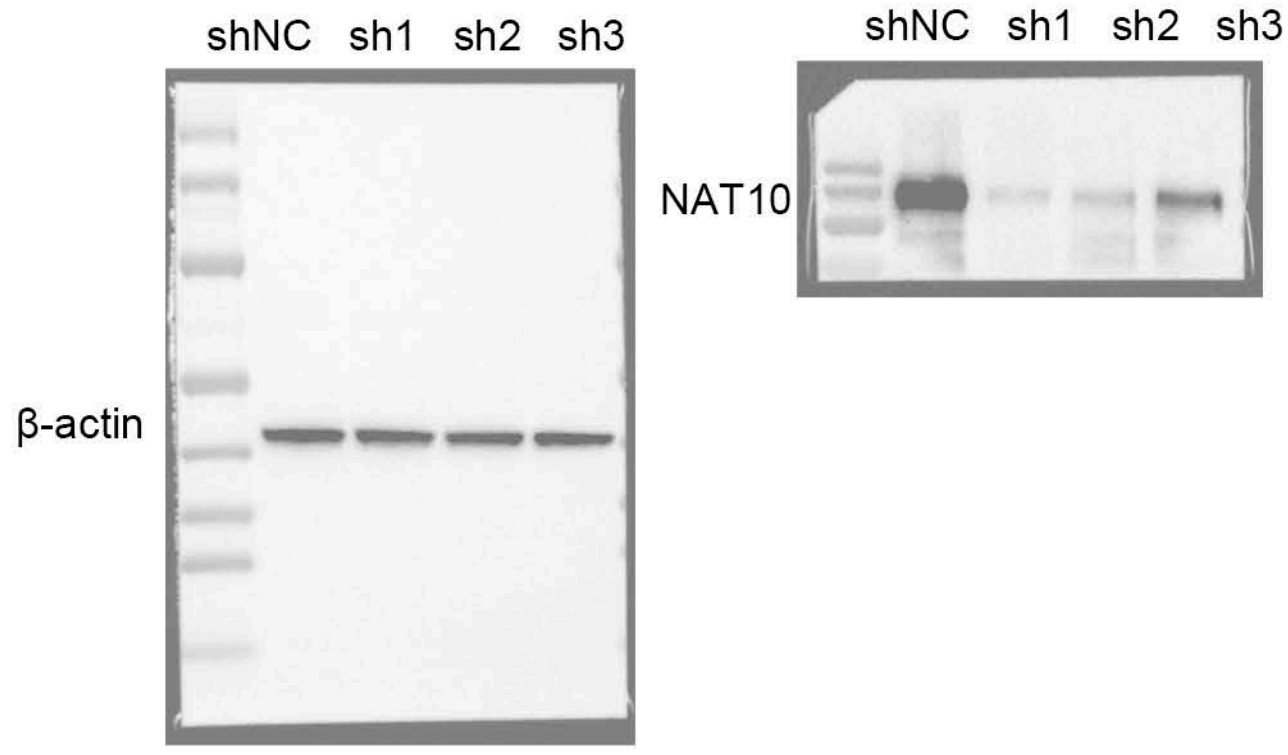

Figure 3H

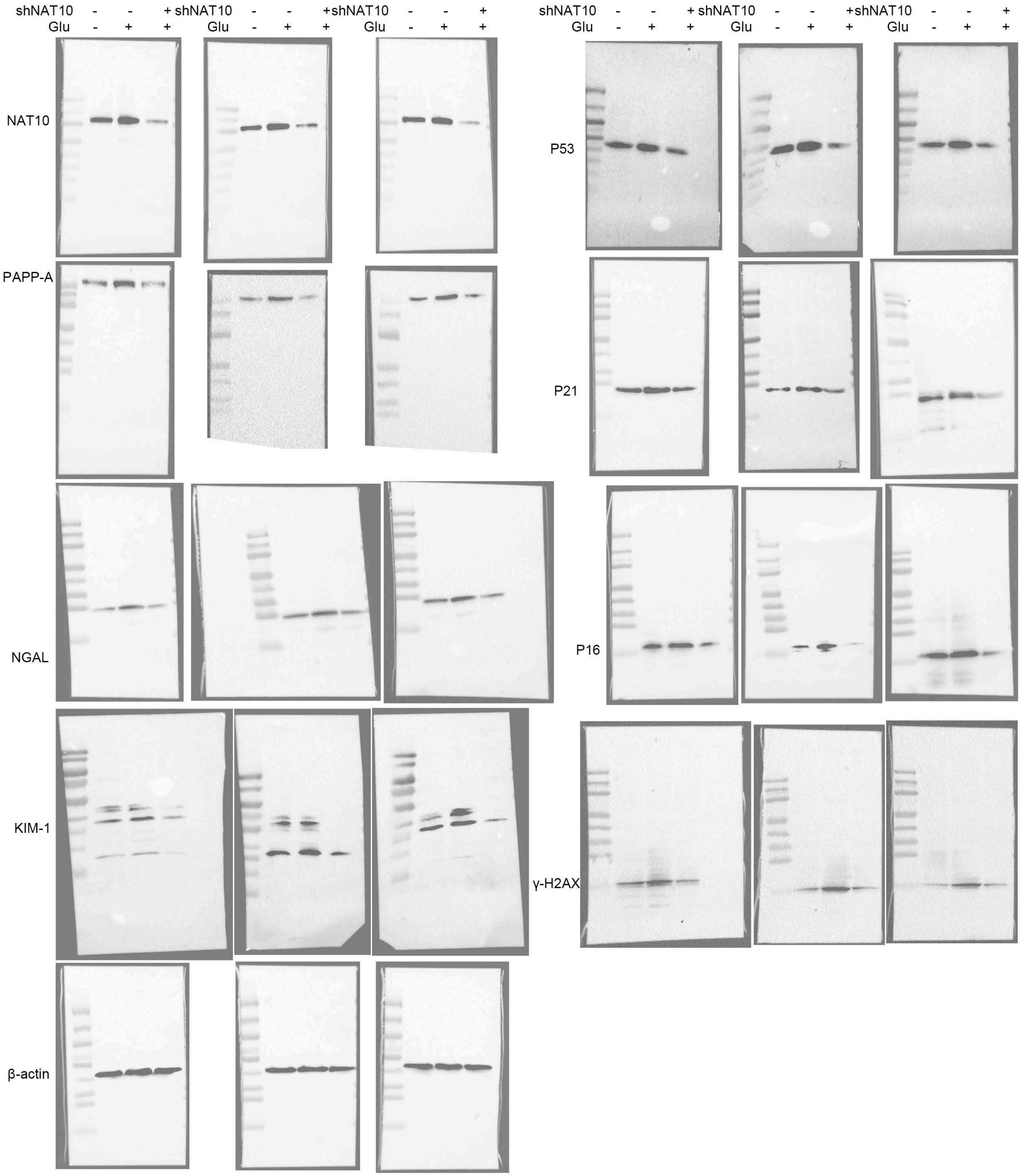

Figure 4C

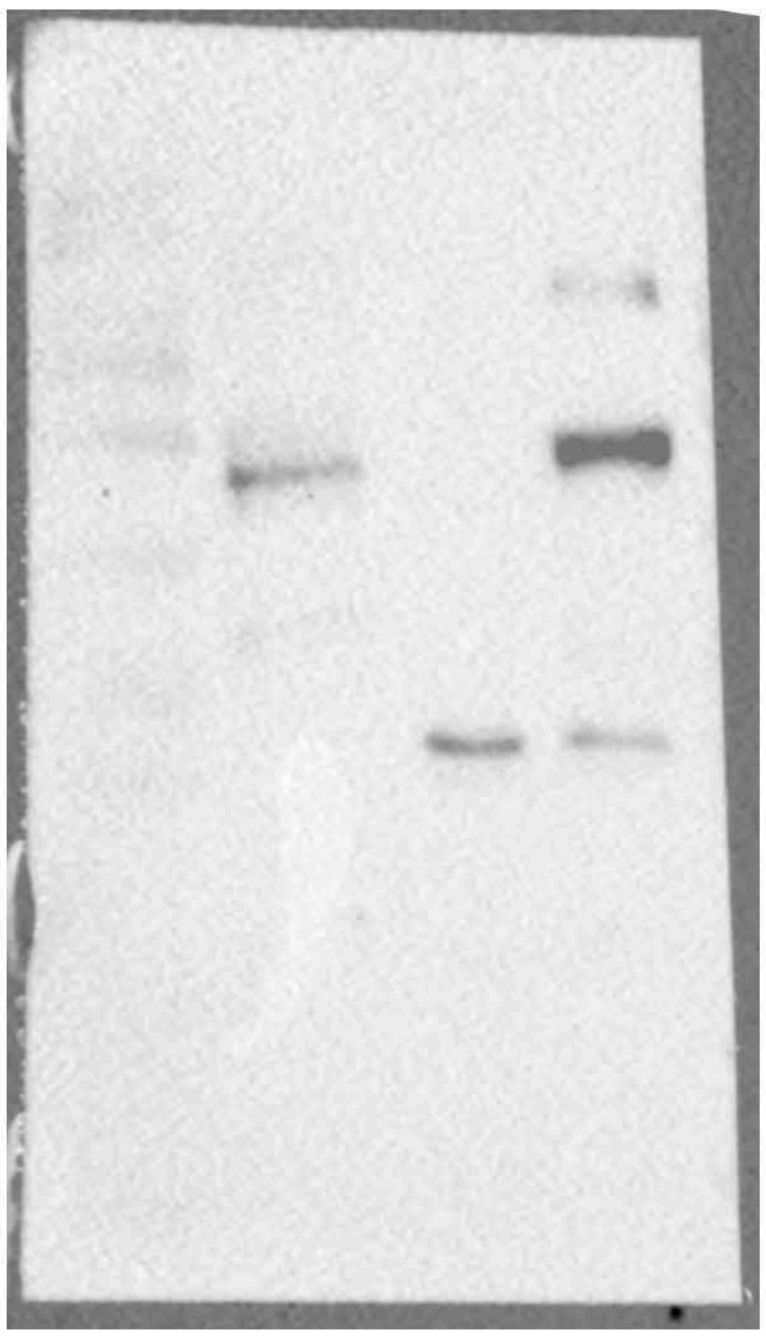

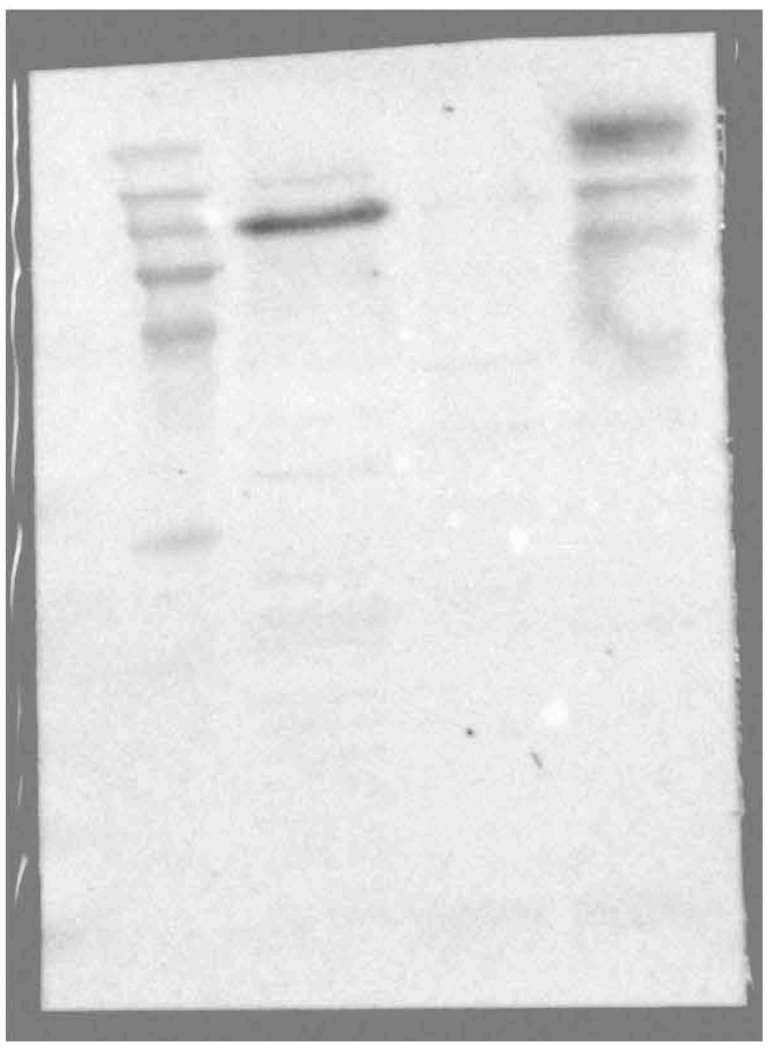

Figure 4B

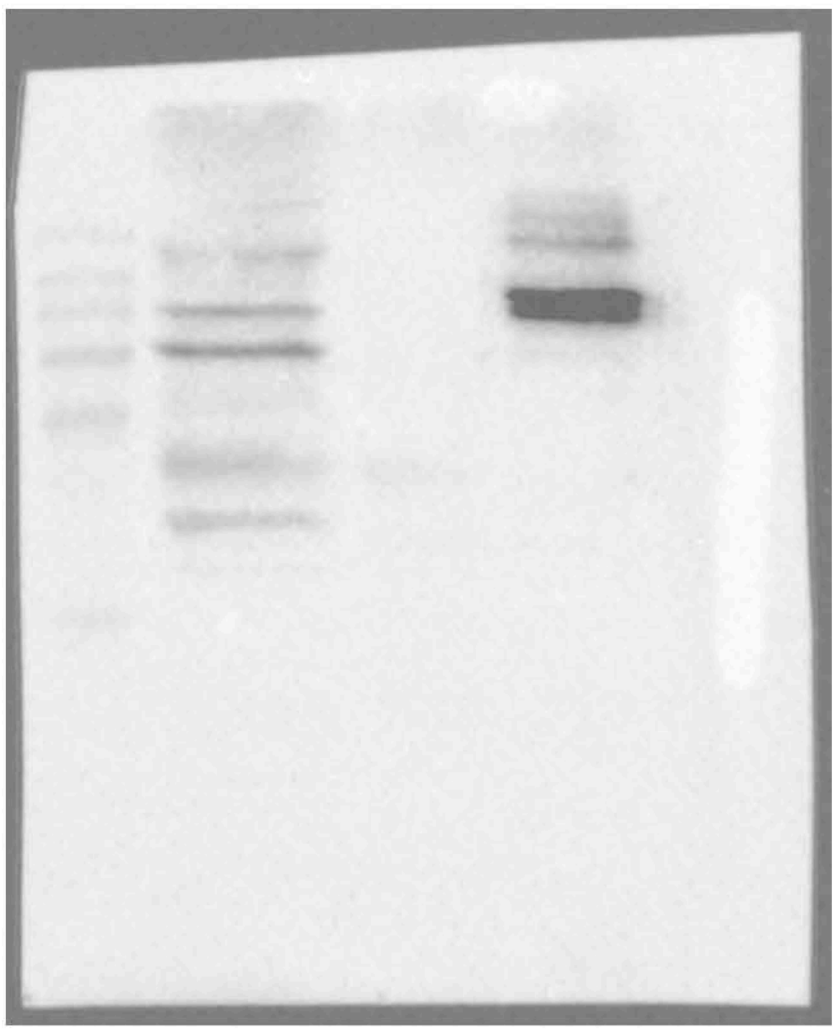

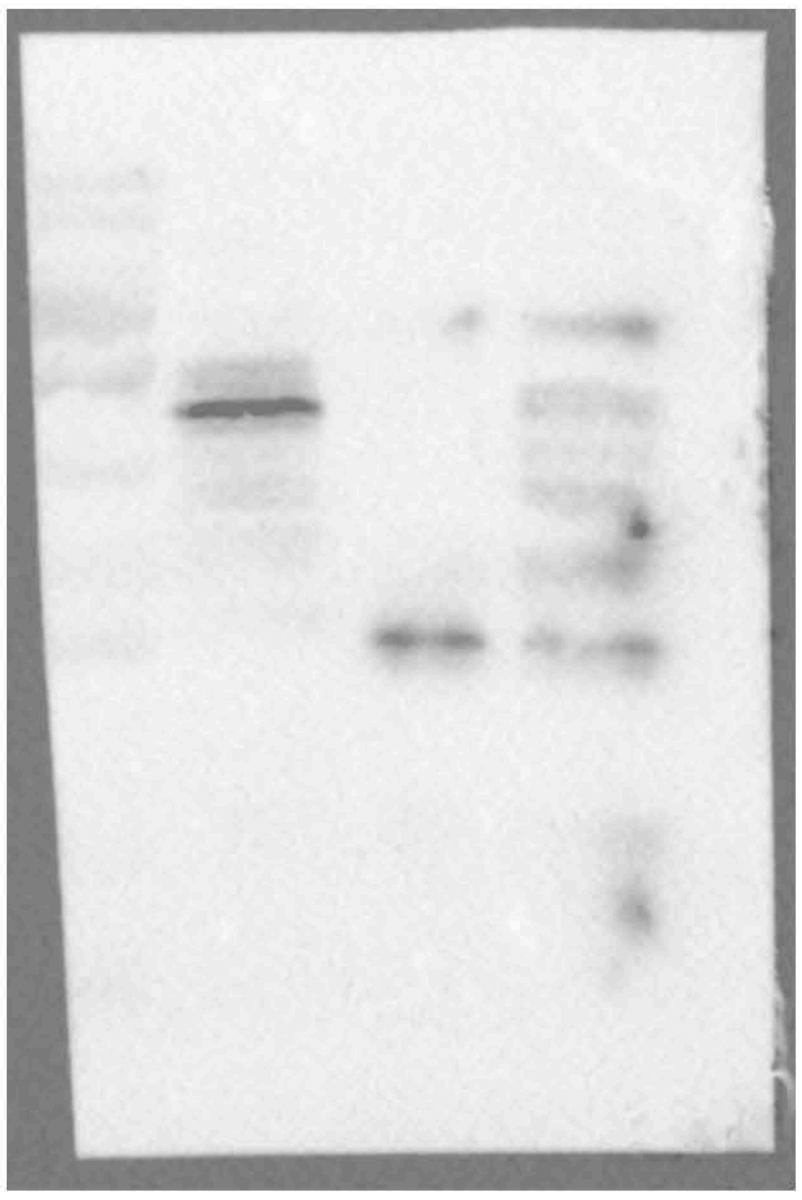

Figure 4D

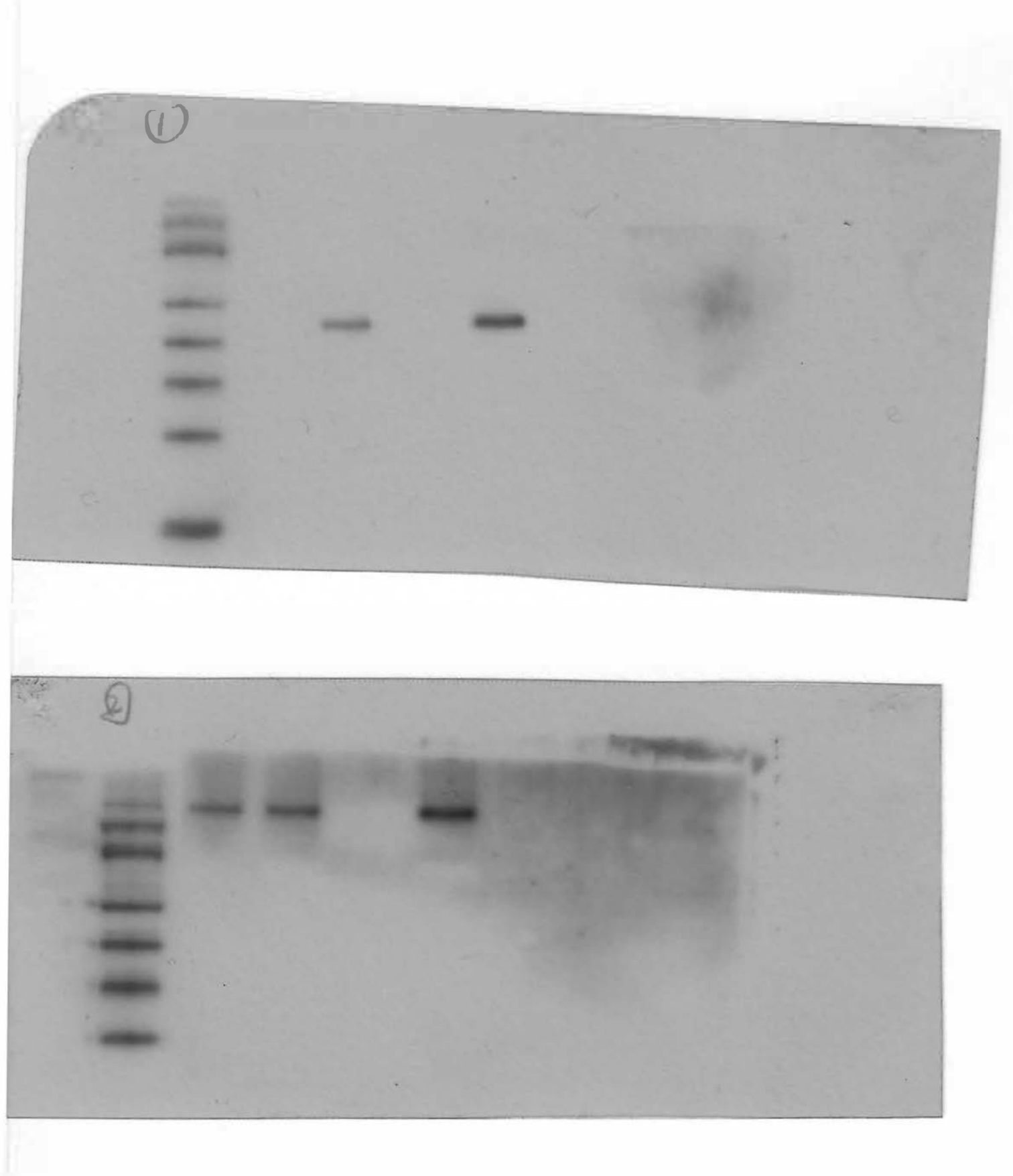

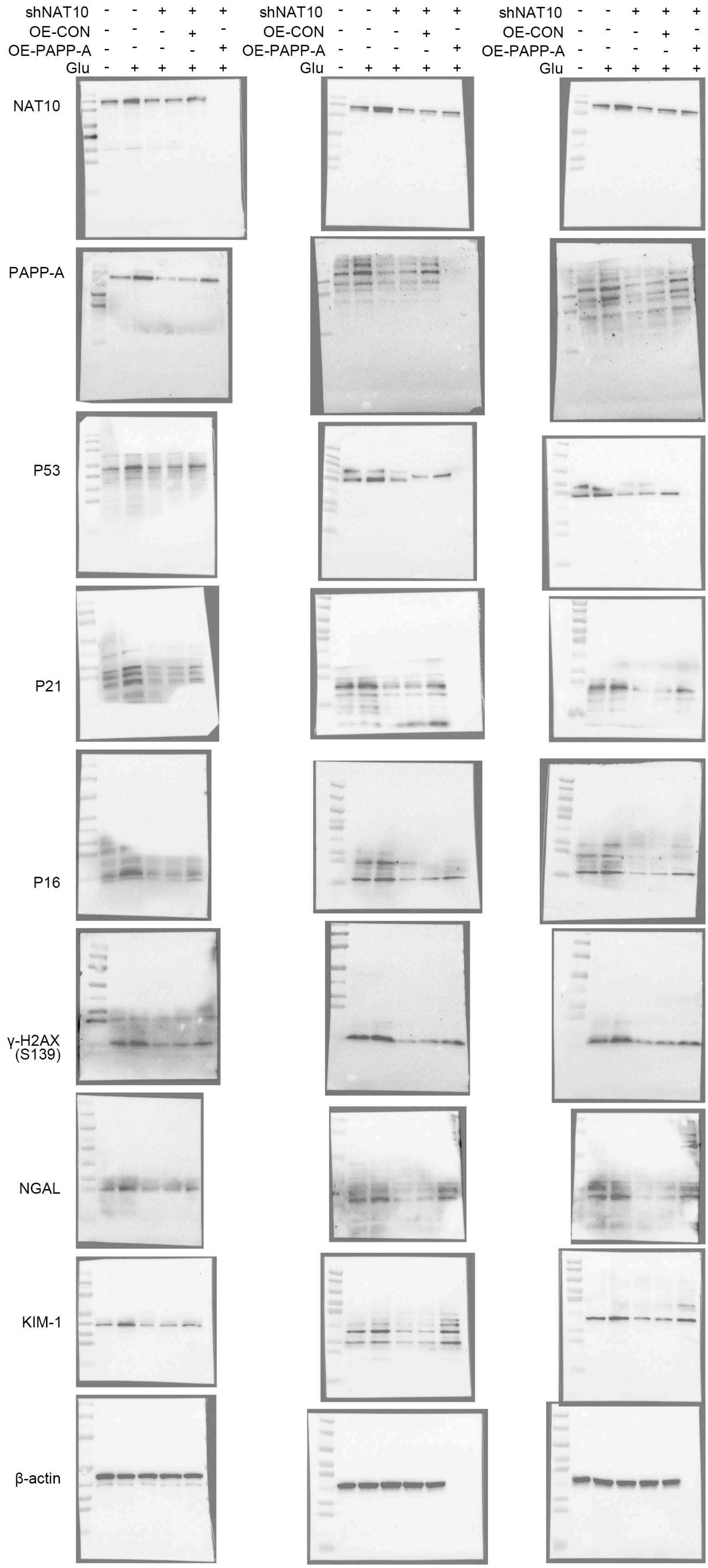
shNAT10 - - + + +
OE-CON - - - + -
OE-PAPP-A - - - - +
Glu - + + + +
NAT10
PAPP-A
P53
P21
P16
γ-H2AX (S139)
NGAL
KIM-1
β-actin

Figure 4E

Figure 5H

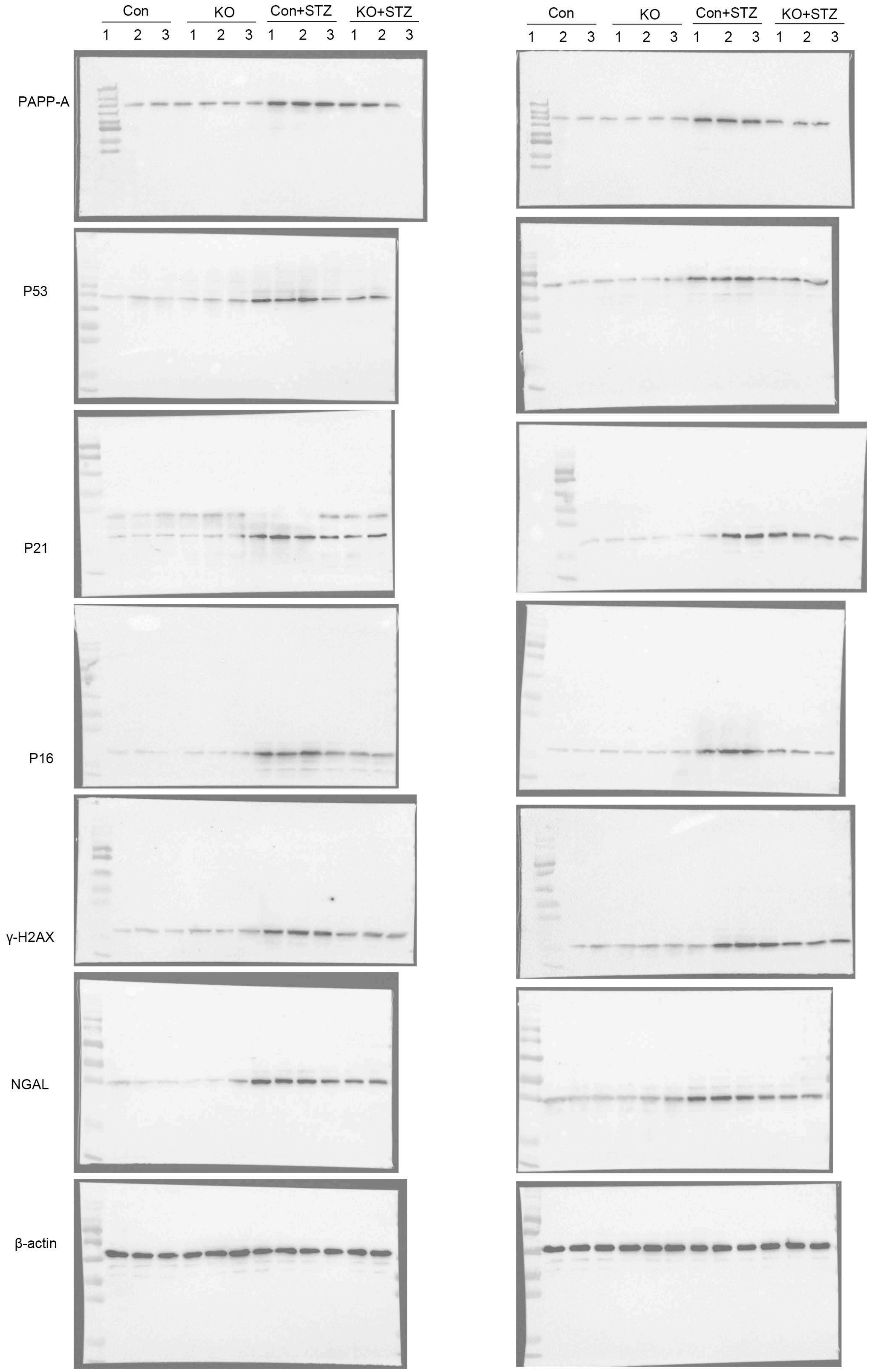

Con
KO
Con+STZ
KO+STZ
1 2 3 1 2 3 1 2 3 1 2 3
PAPP-A
P53
P21
P16
γ-H2AX
NGAL
β-actin

Figure 6E

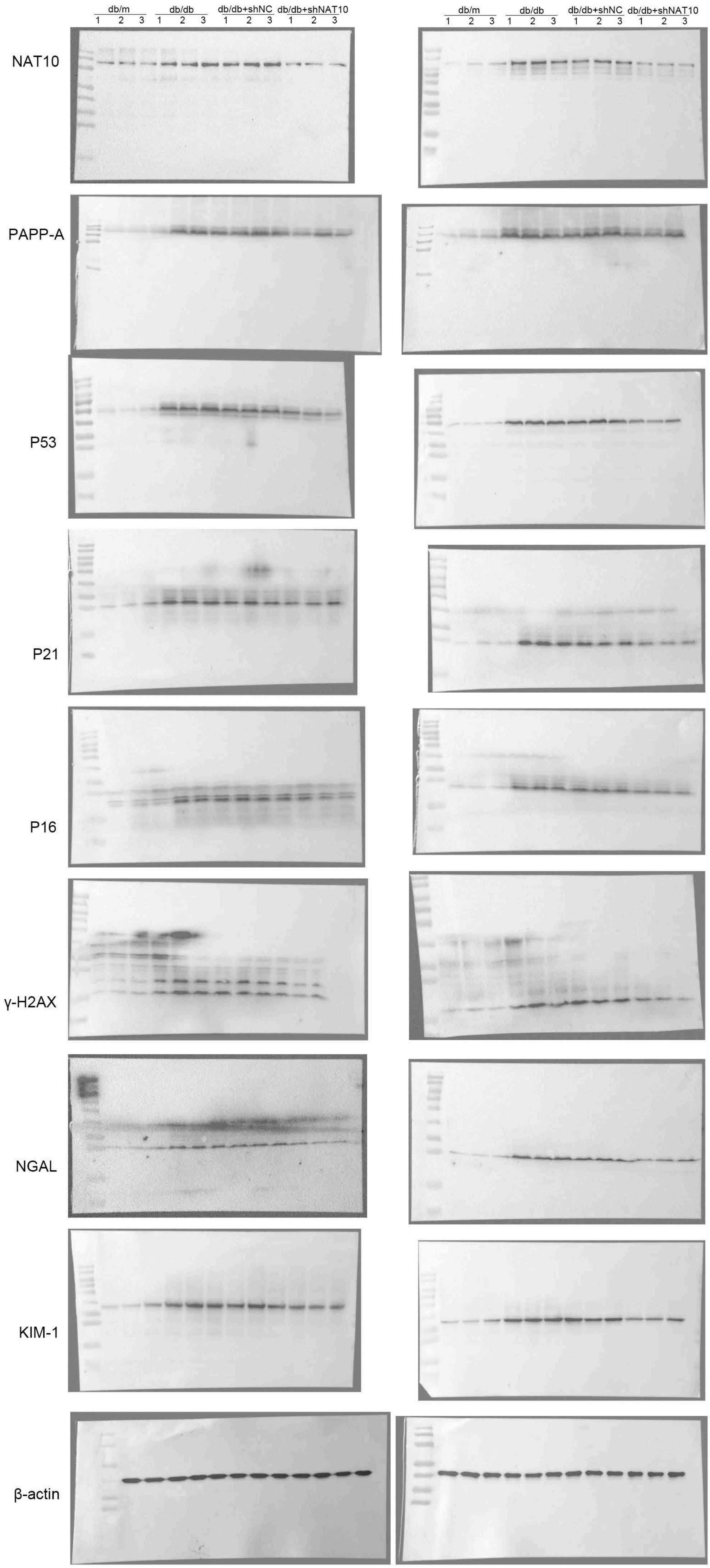

db/m
db/db
db/db+shNC
db/db+shNAT10
1 2 3 1 2 3 1 2 3 1 2 3
NAT10
PAPP-A
P53
P21
P16
γ-H2AX
NGAL
KIM-1
β-actin

Figure 7E

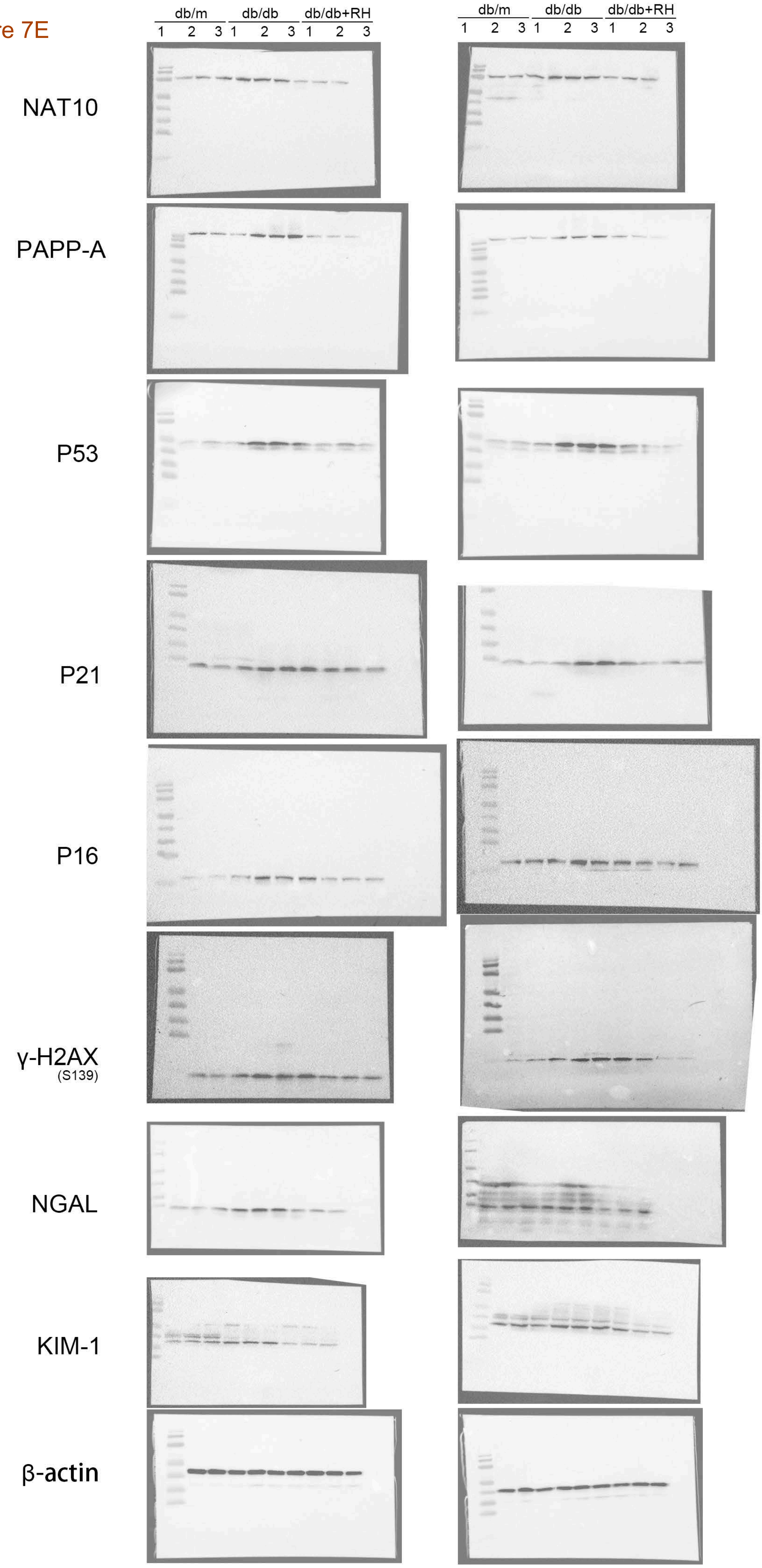

db/m
db/db
db/db+RH
1 2 3 1 2 3 1 2 3
db/m
db/db
db/db+RH
1 2 3 1 2 3 1 2 3
NAT10
PAPP-A
P53
P21
P16
γ-H2AX
(S139)
NGAL
KIM-1
β-actin

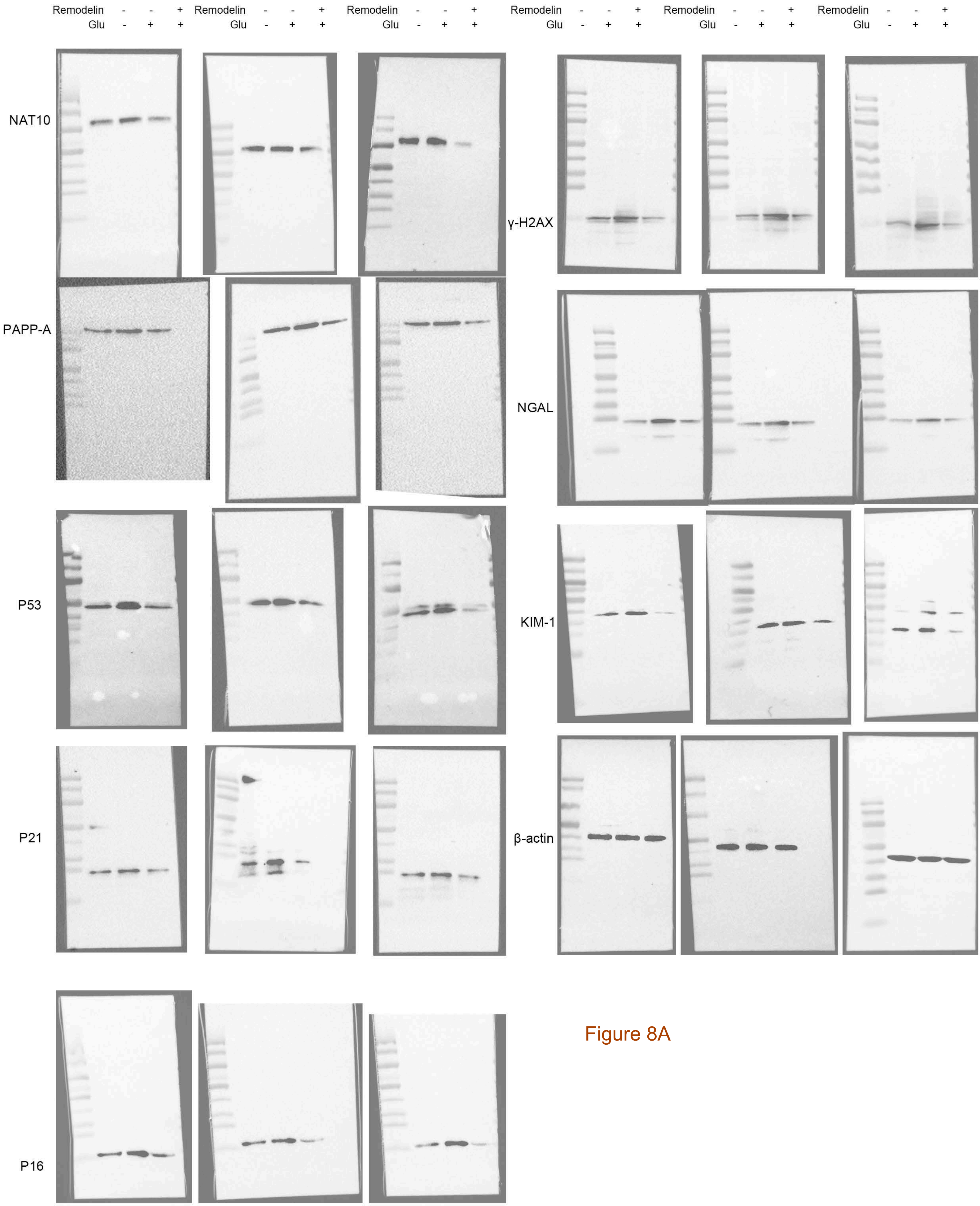
Remodelin - - +
Glu - + +
Remodelin - - +
Glu - + +
Remodelin - - +
Glu - + +
Remodelin - - +
Glu - + +
Remodelin - - +
Glu - + +
Remodelin - - +
Glu - + +
NAT10
γ-H2AX
PAPP-A
NGAL
P53
KIM-1
P21
β-actin
P16

Figure 8A

S-Figure 3C

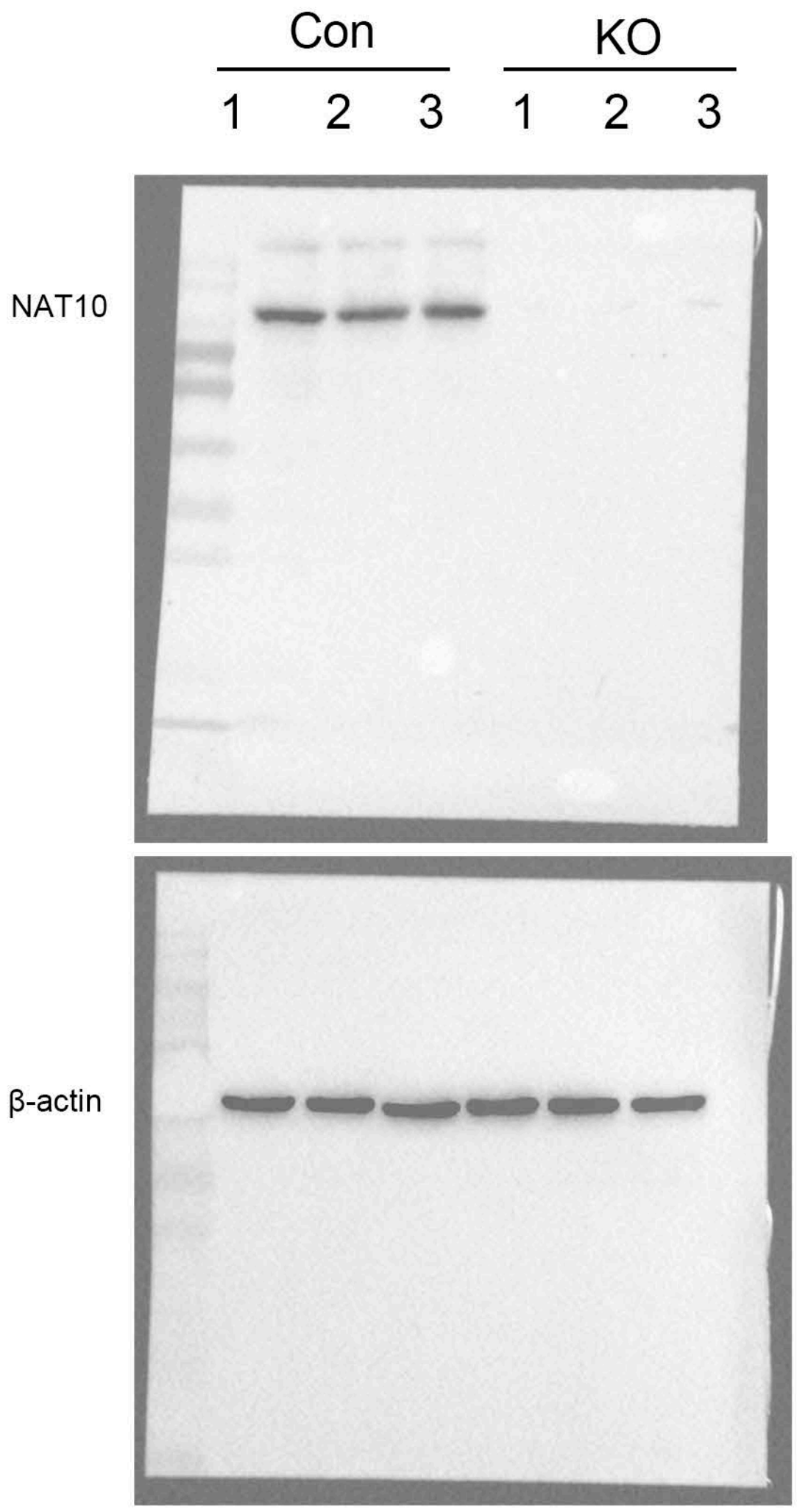

S-Figure 7B

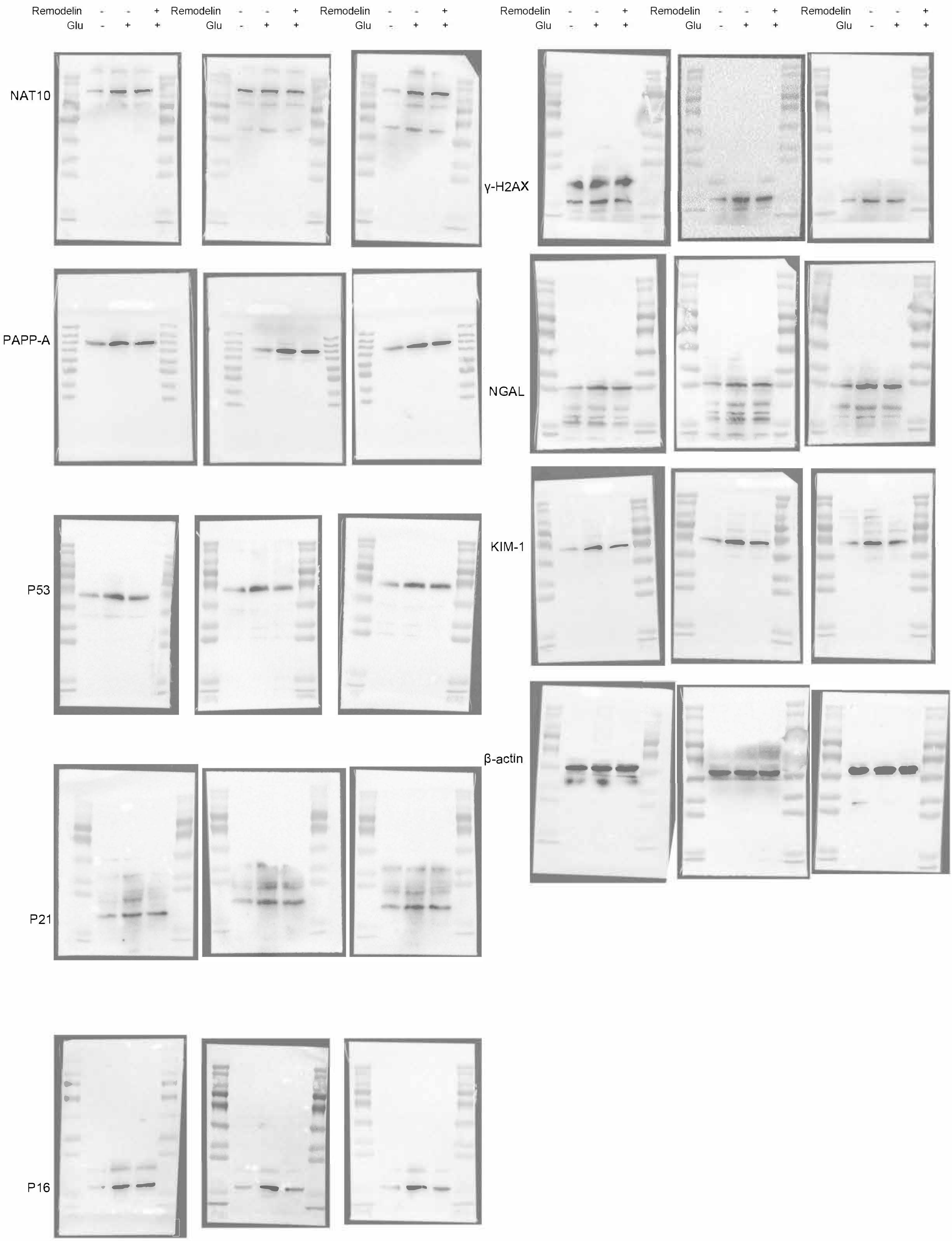
Remodelin - - +
Glu - + +
NAT10
PAPP-A
P53
P21
P16
γ-H2AX
NGAL
KIM-1
β-actin
